# Oral Anticoagulation Timing in Patients with Acute Ischemic Stroke and Atrial Fibrillation

**DOI:** 10.1055/a-1669-4987

**Published:** 2021-12-31

**Authors:** Po-Yin Chang, Wei-Ting Wang, Wei-Lun Wu, Hui-Chin Chang, Chen-Huan Chen, Yi-Wen Tsai, Shih-Hwa Chiou, Gregory Y. H. Lip, Hao-Min Cheng, Chern-En Chiang

**Affiliations:** 1 Office of Surveillance and Epidemiology, Center for Drug Evaluation and Research, U.S. Food and Drug Administration, Maryland, United States; 2 Division of Cardiology, Department of Medicine, Taipei Veterans General Hospital, Taipei City, Taiwan; 3 Department of Medicine, National Yang Ming Chiao Tung University, Taipei, Taiwan; 4 School of Medicine, National Yang Ming Chiao Tung University, Taipei, Taiwan; 5 Institute of Clinical Medicine, National Yang Ming Chiao Tung University, Taipei, Taiwan; 6 Institute of Health and Welfare Policy, National Yang Ming Chiao Tung University, Taipei, Taiwan; 7 Department of Medical Education, Taipei Veterans General Hospital, Taipei, Taiwan; 8 Institute of Public Health, National Yang Ming Chiao Tung University, Taipei, Taiwan; 9 Liverpool Centre for Cardiovascular Science, University of Liverpool and Liverpool Heart and Chest Hospital, Liverpool, United Kingdom; 10 Aalborg Thrombosis Research Unit, Department of Clinical Medicine, Aalborg University, Aalborg, Denmark; 11 Center for Evidence-based Medicine, Taipei Veterans General Hospital, Taipei, Taiwan; 12 General Clinical Research Center, Taipei Veterans General Hospital, Taipei, Taiwan

**Keywords:** atrial fibrillation, acute ischemic stroke, oral anticoagulants, timing, stroke recurrence

## Abstract

**Background and Purpose** Oral anticoagulants (OACs) prevent stroke recurrence and vascular embolism in patients with acute ischemic stroke (AIS) and atrial fibrillation (AF). Based on empirical consensus, current guidance recommends a “1–3–6–12 days” rule to resume OACs after AIS. This study investigated the suitability of guideline-recommended timing for OAC initiation.

**Methods** Using data of 12,307 AF patients hospitalized for AIS, for the period 2012 to 2016, in Taiwan's National Health Insurance Research Database, we constructed a sequence of cohorts of OAC users and propensity score-matched nonusers, creating one cohort on each day of OAC initiation for 30 days since admission. Composite outcome included effectiveness (cardiovascular death, ischemic stroke, myocardial infarction, transient ischemic attack, systemic embolism, and venous thromboembolism) and safety (intracranial hemorrhage, gastrointestinal bleeding, and hematuria) outcomes. Comparing with nonusers, we examined the risks in the early OAC use (within 1–3–6–12 days) or guideline-recommended delayed use. Indirect comparison between the early and delayed use was conducted using mixed treatment comparison.

**Results** Across the AIS severity, the risks of composite or effectiveness outcome were lower in OAC users than nonusers, and the risks were similar between the early and delayed use groups. In patients with severe AIS, early OAC use was associated with an increased risk of safety outcome, with a hazard ratio (HR) of 1.67 (confidence interval [CI]: 1·30–2·13) compared with nonusers and a HR of 1.44 (CI: 0·99–2·09) compared with the delayed use.

**Conclusion** Our study findings support an early OAC initiation in AF patients with mild-to-moderate AIS and a routine delayed use of OACs can be considered in those with severe AIS to avoid a serious bleeding event.

## Introduction

Stroke is a leading cause of mortality and disability, resulting in substantial economic costs in terms of poststroke care. 1 Cardioembolic strokes, most frequently caused by atrial fibrillation (AF), 2 are found to be related to worse outcomes compared with other non-AF-related strokes.

Lifelong use of oral anticoagulants (OACs) has been recommended for secondary stroke prevention. 3 4 However, the optimal timing to resume OAC in AF patients with acute ischemic stroke (AIS) remains a clinical challenge. Early non-vitamin K antagonist OACs (NOACs) within 2 days of AIS had been shown to be associated with a 5% rate of hemorrhagic transformation, 5 whereas a delayed initiation may leave the patients at an increased risk of recurrent ischemic stroke. The 2018 European Heart Rhythm Association practical guide proposed a “1–3–6–12 days rule” to resume OAC after an AIS in patients with AF, 4 6 based on expert consensus opinion without supporting evidence from large-scale randomized controlled trials (RCTs). Recently, one meta-analysis of individual-level data from seven prospective observational studies, including CROMIS-2, 7 RAF, 8 RAF-NOACs, 9 SAMURAI, 10 NOACISP, 11 Erlangen, 12 and Verona 13 registry, suggested that that early NOAC treatment after AIS, when compared with vitamin K antagonist (VKA), was associated with a reduced risk of intracranial hemorrhage (ICH). 14 Interestingly, Mizoguchi and colleagues compared the early (≤3 days) with the delayed (≥4 days) initiation of NOACs after AIS or transient ischemic attack (TIA) in the SAMURAI study and did not observe a difference in risks of stroke, major bleeding, and death between the groups. 15 The study by Mizoguchi and coauthors, however, did not address the potential immortal-time bias because patients who initiated NOACs in the delayed period, by definition, had to be alive and free of ischemic stroke in the early period (and thus to be “immortal” to outcomes of interest.)

Four ongoing RCTs, including ELAN (NCT03148457, Switzerland), OPTIMAS (EudraCT, 2018003859–38, United Kingdom), TIMING (NCT02961348, Sweden), and START (NCT03021928, United States), are to determine the optimal time for initiating OACs after AIS. However, these RCTs only compare the early and delayed initiation of NOACs with fixed intervals, without stratified randomization based on prespecified AIS severity, except ELAN stratifies patients based on the size of the infarction. Moreover, these RCTs fail to investigate the comparative effectiveness and safety of VKA in patients with AIS secondary to AF.

The present study aimed to examine the benefit and risk of early and delayed use of OACs, including NOACs and VKAs, in AF patients hospitalized for AIS. Immortal-time bias is a challenging issue in comparing different strategies of treatment initiation in observational studies, and we constructed a sequence of stroke severity-specific cohorts with propensity score (PS) matching to reduce immortal-time bias and confounding bias. The study results could provide real-world evidence of the optimal timing to initiate OACs after an AIS event among patients with AF.

## Methods

### Taiwan National Health Insurance Research Databases

Taiwan initiated its single-payer, universal National Health Insurance program in March 1995. Enrolment is mandatory. As of 2020, membership consisted of approximately 23,622,000 individuals (99·9% of Taiwan's population). The National Health Insurance Research Database (NHIRD) captures all medical claims, including disease diagnoses, procedures, and prescription fills in the records of inpatient, outpatient, and emergency visits since 2000 for research purposes. The consistency, reliability, and disease diagnostic accuracy of the NHIRD for research in cardiovascular, bleeding, and mortality outcomes among patients with AF and/or AIS have been validated. 16 17 18 19 20 21 22 23 The Institutional Review Board of the National Yang-Ming University, Taiwan, approved this research study (YM104104E).

### Study Population

**Fig. 1:**
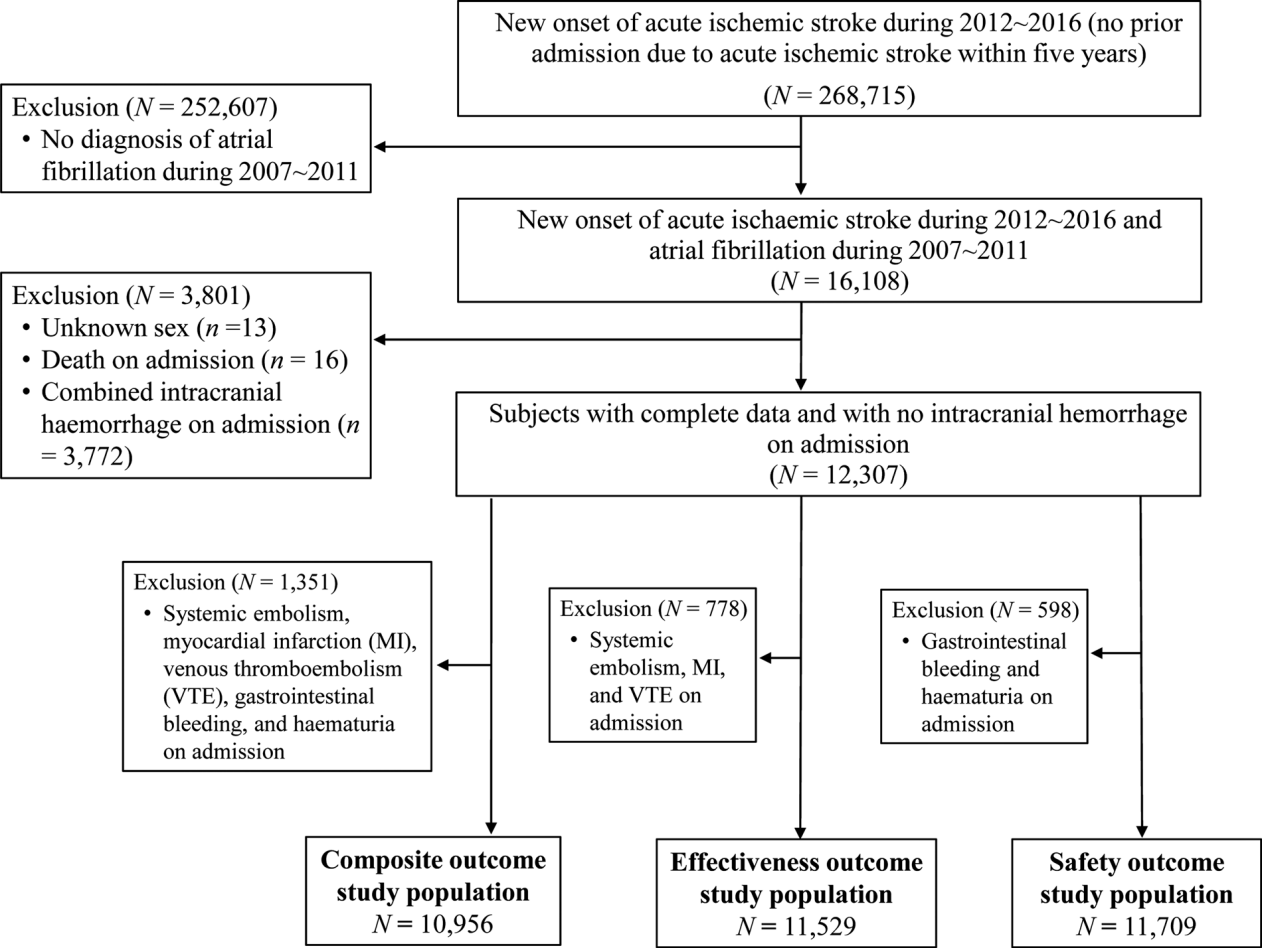
Flowchart of patient selection. AIS, acute ischemic stroke.

The base cohort included 268,715 patients who presented a new AIS (“index stroke event”) from January 1, 2012 to December 31, 2016, who did not have a diagnosis of hemorrhagic stroke or TIA on the admission day, and who did not have any inpatient diagnosis of ischemic stroke within 5 years before the index stroke event ( Fig. 1 ). Using algorithms validated in the NHIRD, AIS was validated by the noncontrast computed tomography (CT) or magnetic resonance imaging (MRI), 21 and AF was defined as having at least one inpatient or outpatient record of the International Classification of Diseases (ICD)-9 or ICD-10 diagnosis code for AF as the primary diagnosis, or having at least two records of AF diagnosis as the secondary diagnosis within 5 years before the index stroke event 23 24 ( Supplementary Table S1 , available in the online version). The final study population consisted of 12,307 AF patients with a new AIS, after excluding patients who lacked AF diagnosis in 2007 to 2011 ( *n* = 252,607), were of unknown sex ( *n* = 13), died on admission ( *n* = 16), or had an ICH diagnosis on admission ( *n* = 3,772).

### Severity of Acute Ischemic Stroke

We calculated a validated stroke severity index (SSI) to categorize the index stroke event into mild (SSI ≤ 5), moderate (5 < SSI ≤ 12), and severe (SSI >12) stroke. 25 26 27 Stroke severity assessed by the National Institutes of Health Stroke Scale (NIHSS) cannot be captured in the administrative claims data, and SSI was closely correlated with NIHSS 27 and performed well in 30-day and 1-year mortality prediction in validation studies. 26 SSI calculation was based on the number of the following tests on admission: airway suctioning, bacterial sensitivity test, general ward stay, intensive care unit stay, nasogastric intubation, osmotherapy (mannitol or glycerol), and urinary catheterization. 27

### Guideline-Recommended OAC Use in AF Patients Hospitalized for Stroke

Guidelines recommend initiation of OACs, including NOACs or warfarin, on the 4th day of admission for a minor AIS, 7th day for a moderate AIS, and 13th day for a severe AIS. 4 6 Depending on the stroke severity, “early use” referred to initiation of OACs within 3 (minor AIS), 6 (moderate AIS), or 12 days (severe AIS) of admission; “delayed use” referred to initiation of OACs between the guideline-recommended initiation day and the 30th day of admission. On a specific day, the exposed group included patients who initiated OACs and the unexposed group included patients who did not initiate OACs. Information of NOAC or warfarin initiation was based on prescriptions in inpatient and outpatient settings. Within 30 days of admission, patients who had prescriptions of NOAC only or VKA only were categorized into the “NOAC group” or the “VKA group,” respectively, and patients who had prescriptions of both NOAC and VKA were categorized into the “both group.”

### Composite Outcome of Effectiveness and Safety

The primary outcome was the first occurrence of a composite outcome of an effectiveness or a safety event. Effectiveness outcomes included ischemic stroke, myocardial infarction (MI), TIA, systemic embolism, venous thromboembolism (VTE), and cardiovascular death. Safety outcomes included ICH, gastrointestinal (GI) bleeding, and hematuria. These outcome events were identified using ICD-9 or ICD-10 diagnosis codes in inpatient records based on validated algorithms in the NHIRD 16 18 19 20 21 22 23 28 ( Supplementary Table S1 , available in the online version).

### Immortal-Time Bias and the Sequence of Cohorts with PS-Matching on Each Day of OAC Initiation

**Fig. 2:**
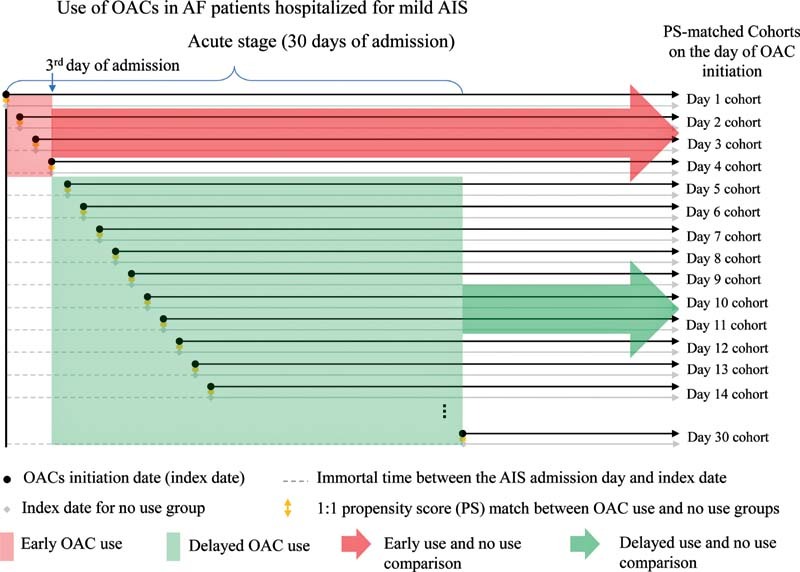
A sequence of propensity-score matched cohorts on each day of OAC initiation from the first to the 30th day of admission, using AF patients with mild AIS as an example. AF, atrial fibrillation; AIS, acute ischemic stroke; OAC, oral anticoagulant.

Immortal time refers to a period of cohort follow-up when study subjects cannot have outcome(s) because of exposure definition. For example, patients who initiated an OAC on the sixth day of admission had to be alive and cannot develop any outcome from the first to the sixth day. Additionally, patients may initiate OACs on a specific day based on physicians' decisions, patients' clinical status, and possibly guideline suggestions. 6 Consequently, the early and delayed use groups likely have different baseline risks of outcomes, and a direct comparison between the two groups could introduce immortal-time bias and confounding bias. 29 30 To reduce these biases, we constructed a sequence of PS-matched cohorts of OAC users and nonusers, creating one cohort on each day of OAC initiation for 30 days since admission ( Fig. 2 ). 30 The day of OAC initiation was defined as the index date for each cohort. Across the three categories of stroke severity and the 30 possible days of OAC initiation, we constructed 90 PS-matched cohorts nested within the study population ( *n* = 12,307) for each of the composite outcome, effectiveness outcome, and safety outcome. The analytic sample of AF patients eligible for PS-matching included 10,956 patients for the composite outcomes, 11,529 patients for the effectiveness outcomes, and 11,709 patients for the safety outcomes ( Fig. 1 ).

Follow-up for each PS-matched cohort started from the cohort index date until the first occurrence of a composite outcome event, noncardiovascular death, loss to follow-up, initiation of OACs in an unexposed group, or December 31, 2017.

### Statistical Analyses

On each of the cohort index dates, we calculated PS using a logistic regression model that included age, sex, use of medication (antihypertensive drugs, antidiabetic drugs, lipid-lowering drugs, OACs, antiplatelets, nonsteroid anti-inflammatory drugs), and medical history of liver disease, peptic ulcer, hypertension, dyslipidemia, ischemic heart disease, ICH, TIA, alcohol intoxication, GI bleeding, hematuria, VTE, systemic embolism, congestive heart failure (CHF), MI, peripheral vascular disease (PVD), cerebrovascular accident, diabetes mellitus, and chronic kidney disease (CKD). We 1:1 matched OAC users to nonusers using the greedy nearest-neighbor technique within a specified caliper width of 0·25 of the standard deviation of the logit of the PS. 31 A nonuser was allowed to be matched to multiple OAC users who initiated OACs on different days. Nonusers could later initiate OACs and become OAC users.

For each level of AIS severity, we pooled all PS-matched cohorts into one analytic sample. 32 Using nonusers as the reference, we performed Cox proportional-hazards models to estimate hazard ratios (HRs) and 95% confidence intervals (CIs) for outcomes in the early use and separately in the delayed use. We stratified Cox models on the index date and adjusted for the PS-matching variables. We used a robust variance estimate to account for within-person correlation. 32

We used the cumulative incidence function 33 to calculate cumulative incidence to account for possible competing risks. 34 Categorical variables were expressed as the number (percentage) and assessed using the Chi-square (χ ^2^ ) or Fisher's exact test.

### Mixed Treatment Comparison

We indirectly compared the risk of outcomes in the early with the delayed use groups using a random-effects model. 35 The indirect comparisons were based on the assumptions of cohort independence and consistency between direct and indirect comparisons. We applied the results comparing the exposed with the unexposed group as direct evidence, and extrapolated the indirect comparison from the direct evidence.

### Net Clinical Benefit Analysis

We performed a net clinical benefit (NCB) analysis, proposed by Singer et al 36 , to examine the risk and benefit profile of early or delayed OAC use, compared with the no use group. The NCB was calculated as: (rate of effectiveness outcome in the no use group – rate of effectiveness outcome in the early [or delayed] use group) − weighting factor × (rate of safety outcome in the early [or delayed] use group – rate of safety outcome in the no use group). The 95% CIs were calculated from rate differences and standard errors estimates using Poisson regression. The weighting factor reflects the relative impact of a safety outcome while receiving an early or a delayed OAC, as opposed to experiencing an effectiveness outcome while not using OACs. We selected three weighting factors (1.5, 2.0, and 3.0) based on publications of the risk and benefit of warfarin use in AF patients. 36 37

## Results

Of the 10,956 patients with AF in the composite outcome analysis ( Supplementary Table S2 , available in the online version), 41·2% ( *n* = 4,513) had a mild stroke, 23·6% ( *n* = 2,582) had a moderate stroke, and 35·2% ( *n* = 3,861) had a severe stroke. Among patients with AF, the proportion of guideline-recommended use of OACs decreased with an increasing stroke severity (early use: 29·0, 26·8, and 21·8%; delayed use: 25·9, 22·2, and 14·5% in mild, moderate, and severe stroke, respectively). Conversely, the proportion of patients with no OAC use increased (45·1, 51·0, and 63·7% in mild, moderate, and severe stroke, respectively).

Across the stroke severity, AF patients who did not initiate OACs after an AIS tended to be older and have more comorbidities than those who did ( Table 1 , Supplementary Tables S3–S13 , available in the online version). For example, peptic ulcers, hypertension, ICH, GI bleeding, CHF, MI, PVD, diabetes, and CKD were more common in AF patients with mild stroke in the no use group than those in the early or delayed OAC use group (all *p* -values before PS-matching: <0·05; Table 1 ). Patients in the no use group were more likely to use antiplatelet therapy and less likely to use OAC at baseline than those in the OAC use groups ( *p* -values before PS-matching: <0·05; Table 1 ). After PS-matching, differences between the OAC use and no use groups disappeared.

**Table 1:** Baseline characteristics of AF patients with mild stroke before and after PS-matching in the analytic sample for composite outcome

	Unmatched cohort ( *n* = 4,513)					PS-matched cohorts					
	Early use ( *n* = 1,307)	Delayed use ( *n* = 1,171)	No use ( *n* = 2,035)	*p* -Value a	*p* -Value b	Early use ( *n* = 1,307)	No use ( *n* = 1,307)	*p* -Value	Delayed use ( *n* = 1,171)	No use ( *n* = 1,171)	*p* -Value
Gender				0·308	0·224			0·694			0·706
Female	597 (45·7)	488 (41·7)	893 (43·9)			597 (45·7)	587 (44·9)		488 (41·7)	497 (42·4)	
Male	710 (54·3)	683 (58·3)	1,142 (56·1)			710 (54·3)	720 (55·1)		683 (58·3)	674 (57·6)	
Age				**<0·001**	**<0·001**			**<0·001**			0·991
≥80	433 (33·1)	383 (32·7)	921 (45·3)			433 (33·1)	436 (33·4)		383 (32·7)	380 (32·5)	
70–80	470 (36·0)	426 (36·4)	654 (32·1)			470 (36·0)	482 (36·9)		426 (36·4)	428 (36·5)	
< 70	404 (30·9)	362 (30·9)	460 (22·6)			404 (30·9)	389 (29·8)		362 (30·9)	363 (31·0)	
Medication use											
Antihypertensive	1,158 (88·6)	993 (84·8)	1767 (86·8)	0·131	0·109	1,158 (88·6)	1,146 (87·7)	0·468	993 (84·8)	996 (85·1)	0·862
Antidiabetic	366 (28·0)	298 (25·5)	601 (29·5)	0·341	**0·013**	366 (28·0)	376 (28·8)	0·664	298 (25·5)	300 (25·6)	0·924
Lipid-lowering agents	306 (23·4)	277 (23·6)	425 (20·9)	0·084	0·067	306 (23·4)	300 (22·9)	0·780	277 (23·6)	274 (23·4)	0·883
Anticoagulant	622 (47·6)	271 (23·1)	237 (11·6)	**<0·001**	**<0·001**	622 (47·6)	625 (47·8)	0·906	271 (23·1)	267 (22·8)	0·844
Antiplatelet	601 (46·0)	622 (53·1)	1197 (58·8)	**<0·001**	**0·002**	601 (46·0)	620 (47·4)	0·456	622 (53·1)	587 (50·1)	0·147
NSAIDs	1,307 (100)	1,171 (100)	2,032 (99·8)	1	1	1,307 (100)	1,307 (100)	1	1,171 (100)	1,171 (100)	1
Comorbidities											
Liver disease	179 (13·7)	193 (16·5)	306 (15·0)	0·283	0·277	179 (13·7)	170 (13·0)	0·604	193 (16·5)	174 (14·9)	0·280
Peptic ulcer disease	353 (27·0)	344 (29·4)	706 (34·7)	**<0·001**	**0·002**	353 (27·0)	357 (27·3)	0·860	344 (29·4)	314 (26·8)	0·167
Hypertension	1028 (78·6)	924 (78·9)	1694 (83·2)	**<0·001**	**0·002**	1,028 (78·6)	1,054 (80·6)	0·206	924 (78·9)	931 (79·5)	0·721
Dyslipidemia	454 (34·7)	414 (35·3)	707 (34·7)	0·997	0·726	454 (34·7)	459 (35·1)	0·837	414 (35·3)	392 (33·5)	0·338
IHD	615 (47·1)	547 (46·7)	1021 (50·2)	0·078	0·059	615 (47·0)	626 (47·9)	0·666	547 (46·7)	539 (46·0)	0·740
ICH	26 (2·0)	20 (1·7)	63 (3·1)	0·052	**0·017**	26 (2·0)	28 (2·1)	0·783	20 (1·7)	16 (1·4)	0·501
TIA	94 (7·2)	76 (6·5)	154 (7·6)	0·686	0·255	94 (7·2)	115 (8·8)	0·129	76 (6·5)	69 (5·9)	0·548
Alcohol intoxication	10 (0·8)	10 (0·8)	24 (1·2)	0·244	0·386	10 (0·8)	12 (0·9)	0·668	10 (0·8)	9 (0·8)	0·817
GI bleeding	125 (9·6)	113 (9·6)	277 (13·6)	<0·001	**0·001**	125 (9·6)	131 (10·0)	0·693	113 (9·6)	104 (8·9)	0·521
Hematuria	65 (5·0)	57 (4·9)	94 (4·6)	0·638	0·749	65 (5·0)	68 (5·2)	0·789	57 (4·9)	47 (4·0)	0·316
VTE	27 (2·1)	18 (1·5)	31 (1·5)	0·241	0·975	27 (2·1)	33 (2·5)	0·433	18 (1·5)	18 (1·5)	1
Systemic embolism	29 (2·2)	28 (2·4)	63 (3·1)	0·130	0·247	29 (2·2)	34 (2·6)	0·523	28 (2·4)	22 (1·89)	0·391
CHF	602 (46·1)	448 (38·3)	907 (44·6)	0·398	**<0·001**	602 (46·1)	576 (44·1)	0·306	448 (38·3)	431 (36·8)	0·468
Myocardial infarction	90 (6·9)	68 (5·8)	193 (9·5)	0·008	**<0·001**	90 (6·9)	77 (5·9)	0·298	68 (5·8)	63 (5·4)	0·653
PVD	107 (8·2)	76 (6·5)	200 (9·8)	0·108	**0·001**	107 (8·2)	95 (7·3)	0·379	76 (6·5)	60 (5·1)	0·157
CVA	610 (46·7)	558 (47·6)	953 (46·8)	0·928	0·653	610 (46·7)	590 (45·1)	0·432	558 (47·6)	550 (47·0)	0·740
Diabetes mellitus	458 (35·0)	390 (33·3)	796 (39·1)	0·017	**0·001**	458 (35·0)	463 (35·4)	0·837	390 (33·3)	376 (32·1)	0·537
CKD	361 (27·6)	300 (25·6)	678 (33·3)	<0·001	**<0·001**	361 (27·6)	370 (28·3)	0·694	300 (25·6)	293 (25·0)	0·739
Secondary prevention with OAC use											
NOAC only	354 (27·1)	666 (56·9)	0			354 (27·1)	0		666 (56·9)	0	
Warfarin only	811 (62·0)	449 (38·3)	0			811 (62·1)	0		449 (38·3)	0	
Both	142 (10·8)	56 (4·8)	0			142 (10·9)	0		56 (4·8)	0	
Antiplatelet c	n/a	n/a	n/a			829 (63·4)	829 (63·4)	1	987 (84·3)	979 (83·6)	0·652

Abbreviations: AF, atrial fibrillation; AIS, acute ischemic stroke; CHF, congestive heart failure; CKD, chronic kidney disease; CVA, cerebrovascular accident; GI, gastrointestinal; ICH, intracranial hemorrhage; IHD, ischemic heart disease; NOAC, non-vitamin K antagonist oral anticoagulant; NSAIDs, nonsteroidal anti-inflammatory drugs; OACs, oral anticoagulants; PS-matched, propensity score-matched; PVD, peripheral vascular disease; TIA, transient ischemic attack; VTE, venous thromboembolism.

Note: Data are shown in *n* (%). Statistically significant *p* -values are denoted in bold.

a *p* -Value: comparing early use versus no use.

b *p* -Value: comparing delayed use versus no use.

c Antiplatelet use from the day of AIS admission to the index date (i.e., the day of OAC initiation or matching).

As the stroke severity increased from mild to severe ( Supplementary Table S14 , available in the online version), the incidence of composite outcome increased (444·5 to 928·3 cases per 1,000 person-years), as did the incidence of effectiveness outcome (292·1 to 654·7 cases per 1,000 person-years). The incidence of safety outcome did not vary substantially (149·5 to 196·4 cases per 1,000 person-years).

### Use of OACs versus No Use and the Risk of Outcomes

#### AF Patients with Mild or Moderate Stroke

When compared with no OAC use, the early or delayed use was associated with a decreased risk of composite outcome with HR ranging from 0·73 (95% CI: 0·62–0·85) to 0·82 (95% CI: 0·67–1·00). The OAC use was not associated with an increased risk of safety outcomes ( Table 2 ).

**Table 2:** Hazard ratio and 95% confidence interval for the composite outcome, effectiveness outcome, and safety outcome, in the early OAC use group and in the delayed OAC use group a

Stroke severity	Mild		Moderate		Severe	
OAC use	Early vs. no use	Delayed vs. no use	Early vs. no use	Delayed vs. no use	Early vs. no use	Delayed vs. no use
Composite outcome						
	**0·75 (0·65, 0·87)**	**0·73 (0·62, 0·85)**	**0·73 (0·61, 0·88)**	0·82 (0·67, 1·00)	**0·79 (0·68, 0·92)**	0·89 (0·73, 1·08)
Stratified by:						
NOAC	**0·64 (0·47, 0·85)**	**0·61 (0·49, 0·76)**	**0·59 (0·38, 0·93)**	**0·73 (0·55, 0·97)**	0·77 (0·51, 1·17)	1·10 (0·84, 1·45)
Warfarin	0·89 (0·74, 1·06)	0·88 (0·68, 1·14)	**0·79 (0·63, 1·00)**	0·76 (0·55, 1·06)	0·86 (0·72, 1·03)	**0·61 (0·45, 0·82)**
Effectiveness outcome						
	**0·83 (0·70, 0·98)**	**0·69 (0·58, 0·83)**	0·88 (0·72, 1·07)	0·84 (0·68, 1·03)	**0·82 (0·70, 0·95)**	**0·76 (0·63, 0·92)**
Stratified by:						
NOAC	0·78 (0·56, 1·07)	**0·52 (0·40, 0·68)**	0·93 (0·59, 1·46)	0·82 (0·63, 1·08)	**0·58 (0·39, 0·86)**	1·02 (0·78, 1·32)
Warfarin	0·86 (0·69, 1·06)	0·99 (0·75, 1·30)	0·95 (0·75, 1·20)	0·80 (0·56, 1·14)	0·98 (0·82, 1·18)	**0·52 (0·39, 0·71)**
Safety outcome						
	0·96 (0·76, 1·22)	**0·75 (0·61, 0·93)**	1·08 (0·80, 1·46)	0·94 (0·69, 1·28)	**1·67 (1·30, 2·13)**	1·16 (0·88, 1·53)
Stratified by:						
NOAC	1·00 (0·58, 1·71)	0·77 (0·58, 1·03)	1·13 (0·61, 2·10)	0·81 (0·51, 1·28)	**2·10 (1·13, 3·92)**	1·18 (0·78, 1·78)
Warfarin	0·95 (0·71, 1·28)	0·73 (0·52, 1·02)	1·07 (0·73, 1·57)	1·11 (0·68, 1·80)	**1·76 (1·29, 2·39)**	1·05 (0·69, 1·59)

Abbreviations: AF, atrial fibrillation; NOAC, non-vitamin K antagonist oral anticoagulant; OACs, oral anticoagulants.

Note: Statistically significant values are denoted in bold.

a A sequence of PS-matched cohorts for each of the three outcomes was constructed on each day from the first to the 30th day of admission. Data of PS-matched cohorts were pooled together for analyses.

#### AF Patients with Severe Stroke

Early use of OACs, compared with no use, was associated with a 0.79-fold (95% CI: 0.68–0.92) risk of composite outcome, 0.82-fold (95% CI: 0.70–0.95) risk of effectiveness outcome, and 1.67-fold (95% CI: 1.30–2.13) risk of safety outcomes. In NOAC- and warfarin-specific analyses, early use of NOAC was associated with a 0·58-fold (95% CI: 0·39–0·86) risk of effectiveness outcome and a 2.10-fold (95% CI: 1·13–3·92) risk of safety outcome. On the contrary, delayed use of warfarin was associated with a 0·52-fold (95% CI: 0.35–0.71) risk of effectiveness outcomes and was not associated with an increased risk of safety outcomes ( Table 2 ).

### Early versus Delayed Use of OACs and the Risk of Outcomes

Across the stroke severity level, the risk of composite or effectiveness outcomes did not significantly differ between the early use and the delayed use groups ( Table 3 ). However, a trend of an increased risk of safety outcomes associated with the early use of OACs was observed, particularly in patients with severe stroke (HR: 1·44, 95% CI: 0·99–2·09, delayed use as reference). In AF patients with severe stroke, the risk of effectiveness outcome was lower in the early use than the delayed use of NOAC (HR: 0·57, 95% CI: 0·35–0·91); the opposite was observed in comparing the early use with the delayed use of warfarin (HR: 1.88, 95% CI: 1.33–2.68).

**Table 3:** Hazard ratio and 95% confidence intervals for the composite outcome, effectiveness outcome, and safety outcome, comparing the early use with the delayed use of OACs in mixed treatment comparison a

Stroke severity	Mild	Moderate	Severe
Composite outcome	1·03 (0·83, 1·27)	0·89 (0·68, 1·17)	0·89 (0·69, 1·14)
Stratified by:			
NOAC	1·05 (0·73, 1·52)	0·81 (0·48, 1·37)	0·70 (0·43, 1·15)
Warfarin	1·01 (0·74, 1·39)	1·04 (0·70, 1·55)	1·41 (0·99, 2·00)
Effectiveness outcome	1·20 (0·94, 1·54)	1·05 (0·79, 1·40)	1·08 (0·85, 1·38)
Stratified by:			
NOAC	1·50 (0·99, 2·28)	1·13 (0·67, 1·92)	**0·57 (0·35, 0·91)**
Warfarin	0·87 (0·61, 1·23)	1·19 (0·78, 1·82)	**1·88 (1·33, 2·68)**
Safety outcome	1·28 (0·93, 1·76)	1·15 (0·75, 1·77)	1·44 (0·99, 2·09)
Stratified by:			
NOAC	1·30 (0·70, 2·40)	1·40 (0·65, 3·01)	1·78 (0·84, 3·75)
Warfarin	1·30 (0·83, 2·04)	0·96 (0·52, 1·79)	1·68 (0·998, 2·82)

Abbreviations: AF, atrial fibrillation; NOAC, non-vitamin K antagonist oral anticoagulant; OACs, oral anticoagulants.

Note: Statistically significant values are denoted in bold.

a Reference group for all mixed treatment comparison was the delayed OAC use.

### Net Clinical Benefit for OAC Use

In patients with mild or moderate stroke, early or delayed use of OACs was associated with a statistically significant NCB, as opposed to no OAC use ( Table 4 ). In patients with severe stroke, use of OACs, compared with no use, was also associated with a NCB although the benefit did not reach statistical significance in the delayed use group.

**Table 4:** Net clinical benefit for the early use and for the delayed use of OACs compared with no use

	Effectiveness outcome			Safety outcome			Net clinical benefit (95% CI) a		
	*n*	Person-year (p-yr)	Incidence (1,000 p-yr)	*n*	Person-year	Incidence (1,000 p-yr)	Weighting factor b		
**1.5**	**2.0**	**3.0**
Mild stroke									
Early use	570	2,009.0	283.7	349	2,493.6	140.0	0.16 (0.09, 0.23)	0.17 (0.08, 0.25)	0.19 (0.08, 0.30)
No use	250	600.6	416.3	119	753.3	158.0	Ref	Ref	Ref
Delayed use	447	1,813.9	246.4	330	2,245.7	146.9	0.10 (0.04, 0.15)	0.11 (0.04, 0.17)	0.13 (0.04, 0.22)
No use	352	1,119.6	314.4	226	1,356.6	166.6	Ref	Ref	Ref
Moderate stroke									
Early use	356	908.6	391.8	190	1,152.0	164.9	0.31 (0.18, 0.45)	0.34 (0.19, 0.49)	0.38 (0.19, 0.58)
No use	179	280.6	638.0	68	323.8	210.0	Ref	Ref	Ref
Delayed use	269	814.2	330.4	170	1,021.3	166.4	0.20 (0.09, 0.30)	0.22 (0.10, 0.34)	0.27 (0.10, 0.44)
No use	203	448.1	453.0	96	445.7	215.4	Ref	Ref	Ref
Severe stroke									
Early use	487	742.9	655.6	219	1,032.3	212.1	0.25 (0.11, 0.38)	0.25 (0.09, 0.40)	0.25 (0.05, 0.44)
No use	330	365.1	903.9	95	450.1	211.1	Ref	Ref	Ref
Delayed use	321	597.8	537.0	141	853.6	165.2	0.12 (−0.01, 0.26)	0.14 (−0.01, 0.29)	0.18 (−0.002, 0.37)
No use	249	412.7	603.3	99	485.0	204.1	Ref	Ref	Ref

Abbreviations: CI, confidence interval; OAC, oral anticoagulant.

a Net clinical benefit was calculated as: (rate of effectiveness outcome in the no use group – rate of effectiveness outcome in the early [or delayed] use group − weighting factor × (rate of safety outcome in the early [or delayed] use group – rate of safety outcome in the no use group), originally proposed by Singer et al. 36

b The weighting factor reflects the relative impact, in terms of death and disability, of a safety outcome while receiving an early or a delayed OAC versus experiencing an effectiveness outcome while not using OACs. The weighting factors were based on those in the publications. 36 37

## Discussion

This study provided the first evidence of a large-scale population and evaluated the effects of early or delayed OAC initiation in a population with AF after AIS by PS-matched cohort on each day since admission, stratified by stroke severity, to overcome immortal-time biases by emulating RCTs. Herein, the key finding was that: first, both early and delayed OAC use could reduce the risk of composite and effectiveness outcomes for each stroke severity. Second, compared with delayed use of OAC, early use, based on the recommendations of current clinical guidelines, was not associated with an excessive risk of composite outcomes. Third, in subjects with severe stroke, early treatment may result in a higher bleeding risk when compared with delayed treatment, despite presenting similar risks for the effectiveness and composite outcomes.

Previous observational studies regarding the timing of OAC initiation after AIS have presented conflicting results. For example, Paciaroni et al have reported that the optimal time to initiate OACs was 4 to 14 days from stroke onset. 8 Similar results have been observed in the RAF-NOAC study, with the lowest composite rates of recurrence and major bleeding for those who initiated NOACs between 3 and 14 days. 9 The 2018 American Heart Association/American Stroke Association guidelines recommended that secondary prevention with OACs should be appropriately instituted within the first 2 weeks, 38 whereas United Kingdom guidelines 39 recommended that OAC administration be deferred until at least 14 days from the onset in patients with disabling ischemic stroke.

However, more recent studies have not supported the 14-day recommendation. Yaghi et al have conducted a registry from eight comprehensive stroke centers and found that OACs started in the 0- to 3-day period were not associated with higher recurrent ischemic events or ICH when compared with those initiated at 4 to 14 days. 40 Clinical Relevance of Microbleeds In Stroke-2 (CROMIS-2) also suggested that early OAC (0–4 days) after AF-related AIS or TIA was not associated with a difference in the composite outcome of stroke, TIA, or death at 90 days, when compared with delayed OAC (≥5 days or never started). 41

Given that the delayed initiation of OAC was not associated with obvious clinical benefits, early use of OACs in mild and moderate AIS patients with AF might be a reasonable alternative. Our finding is consistent with the previous two small randomized trials, 42 43 which provided reassurance regarding the safety of early initiation of administration of rivaroxaban or dabigatran in patients with mild-to-moderate ischemic stroke (NIHSS < 9).

Previous recommendations for the delayed initiation of OAC were based on concerns of hemorrhagic transformation after AIS. However, in our analysis, at least in subjects with mild-to-moderate stroke severity, a delayed OAC use for secondary prevention in patients with AF and AIS is not an evidence-based recommendation and should not be employed as routine clinical practice; however, in severe patients with AIS, delaying the use of OACs may reduce the risk of bleeding events. RCTs are needed to define the appropriate timing of OACs initiation.

One of the major advantages of our study is the large study population from a nationwide cohort, providing the opportunity to perform PS matching with sufficient event numbers for statistical inference. Another important strength is the comprehensive analytic framework in our study, especially the approach in dealing with immortal-time bias. For research questions involving strategies with different timings, immortal-time bias and confounding bias are difficult issues to resolve in observational studies. By utilizing the day-by-day PS-matching approach, we reduced the immortal-time bias and confounding bias when comparing different strategies. Furthermore, in the present real-world cohorts, we employed the novel mixed treatment comparison meta-analysis techniques for indirect comparisons to obtain relative effects of early versus delayed OAC use in AF patients with AIS.

Several limitations of the present study need to be acknowledged. First, selection bias is an inherent limitation of observational studies. Second, the disease status and outcomes were identified by validated algorithms, 16 17 18 19 20 21 22 23 28 which might not represent patients' real conditions as the codes were designed to claim health insurance. Third, there may be residual confounding from unmeasured or unknown covariates as NHIRD was unable to provide laboratory data such as international normalized ratio to evaluate the controlled efficacy of VKAs or imaging data, including CT scan, MRI, and echocardiography, to fully evaluate the clinical status. Claims-based databases also lack the information regarding stroke lesion volume; therefore, the current study applied the validated tool, SSI, 25 26 27 for assessing stroke severity rather than NIHSS. The latter, however, is the most widely accepted tool to assess the severity of stroke. Fourth, our study samples were recruited repeatedly in different cohorts to imitate an RCT design. Our estimated results could be confounded by unsatisfied independence. Owing to similar inclusion criteria, methodology, and controlled variables in our study, the consistency assumption of the indirect comparisons is less concerning. 44 Lastly, the delayed or early initiation of OACs was based on current guideline recommendations, and future studies to determine the most appropriate timing to resume OACs are warranted.

## Conclusion

In patients with AF admitted for AIS, early initiation of OACs and the routine delayed use appeared to result in a comparable risk of composite clinical outcome across the levels of stroke severity. The risk of bleeding events seemed to be similar for all the OAC use groups in patients with mild-to-moderate AIS. However, such a risk was particularly concerning for patients with severe AIS who resumed OACs early. The current study findings support an early OAC initiation in AF patients with mild-to-moderate AIS and a routine delayed use of OACs in those with severe AIS to avoid a serious bleeding event. The optimal timing of OAC initiation after AIS requires further investigation.

## Notes

**What is known about this topic?**

- Evidence has suggested that high-risk patients with atrial fibrillation (AF) should routinely administer lifelong oral anticoagulants (OACs) for secondary stroke prevention.
- The 2018 European Heart Rhythm Association (EHRA) practical guide proposed a “1–3–6–12 days rule” to resume OAC after an acute ischemic stroke in patients with AF, which is an expert consensus opinion, lacking supporting evidence from large-scale randomized controlled trials or real-world observational studies.

**What does this paper add?**

- Both early and delayed OAC uses could reduce composite and effectiveness outcomes for each stroke severity.
- Compared with delayed use of OAC, early use, based on the recommendations of current clinical guidelines, was not associated with an excessive risk of composite outcomes.
- In subjects with severe stroke, early treatment may result in a higher bleeding risk when compared with delayed treatment, despite presenting similar risks for the effectiveness and composite outcomes.
