# Supplementary material for: Oral Anticoagulation Timing in Patients with Acute Ischemic Stroke and Atrial Fibrillation
Source: Thromb Haemost. 2021 Dec 31;122(6):939–50. doi: 10.1055/a-1669-4987 (PMC9251709; doi:10.1055/a-1669-4987)
Supplement: Supplementary file 1 — Supplementary Material [file 10-1055-a-1669-4987-s210330.pdf]

Supplementary Table S1 International Classification of Disease codes used in the study

Outcomes	ICD9 and 10
Effectiveness	1. Ischemic stroke: 433.01, 433.11, 433.21, 433.31, 433.81, 433.91, 434.01, 434.11, 434.91, I63, I69.3<br>2. Cardiovascular death: I10–I15, I01–I02, I05–I09, I20–I25, I27, I30–I52, I60–I71<br>3. Myocardial infarction: 410–412, 429.7, I21–I24, I252<br>4. Systemic embolism: 444, I74<br>5. Venous thromboembolism: 453, 415, 451, 452, 437.6, I82, I26, I80, I81, I63.6, I67.6<br>6. Transient ischemic attack: 435, 437.7, 362.34, G45, H34.0
Safety	1. Hemorrhagic stroke: 430–432,852–853, I60–I62, I69.0–I69.2, S064 × 0A–S066 × 9A, S06340A–S06369A<br>2. Hematuria: 599.7, R31<br>3. Gastrointestinal bleeding:<br>*ICD9*<br>456.0, 530.7, 531.0, 531.2, 531.4, 531.6, 532.0, 532.2, 532.4, 532.6, 533.0, 533.2, 533.4, 533.6, 534.0, 534.2, 534.4, 534.6, 569.3, 578.0, 578.1, 578.9, 531.1, 531.5, 532.1, 532.5, 533.1, 533.5, 534.1, 534.5, 456.20, 530.82, 535.01, 535.11, 535.21, 535.31, 535.41, 535.51, 535.61, 535.71, 537.83, 537.84, 562.02, 562.03, 562.12, 562.13, 569.85, 530.21, 569.86<br>*ICD10*<br>K22.6, K25.0–K25.2, K25.4–K25.6, K26.0–K26.2, K26.4–K26.6, K27.0–K27.2, K27.4–K27.6, K28.0–K28.2, K28.4–K28.6, K62.5, K92.0–K92.2, I85.01, I85.11, K31.82, K29.01, K29.31, K29.41, K29.51, K29.61, K29.21, K29.31, K29.61, K29.71, K29.91, K29.81, K31.82, K57.11, K57.51, K57.01, K57.13, K57.41, K57.53, K57.31, K57.51, K57.91, K57.21, K57.33, K57.41, K57.53, K57.81, K57.93, K55.21, K22.11, K63.81, K31.811
Composite	Combination of effectiveness and safety outcomes
Drugs	ATC code
NOAC	Dabigatran (B01AE07), Rivaroxaban (B01AF01), Edoxaban (B01AF03), Apixaban (B01AF02)
Warfarin	B01AA03
NSAID	M01AB16, M02AA25, A01AD05, B01AC06, N02BA01, M01BA03, N02BA51, B01AC56, N02BA71, C10BX08, C10BX12, C10BX06, C07FX04, N02AJ07, N02AJ02, C07FX03, N02AJ18, C10BX02, C10BX05, C10BX01, C10BX04, C07FX02, C08CA51, L01XX33, M01AH01, D11AX18, M01AB05, M02AA15, S01BC03, S01CC01, M01AB55, N02BA11, M01AB08, M01AH05, M01AE04, M01AG03, M01AE09, M02AA19, R02AX01, S01BC04, N02AJ08, M01AE14, C01EB16, G02CC01, M01AE01, M02AA13, R02AX02, M01AE51, N02AJ19, C01EB03, M01AB01, M02AA23, S01BC01, S01CC02, M01AB51, M01AE17, M02AA27, M01AE03, M02AA10, M01AE53, M02AA28, N02AJ14, M01AB15, S01BC05, S01FB51, M01AG04, M02AA18, M01AG01, M01AC06, M01AC56, M01AX01, G02CC02, M01AE02, M02AA12, M01AE52, M01AE56, M01AX17, M02AA26, M01AA01, M02AA01, M01BA01, M01AC01, M02AA07, S01BC06, M01AH02, A07EC, D02AF, J04AA, M02AC, N02BA, P02DA, J04AA01, D01AE12, S01BC08, N02BA06, M01AB02, M01AC02, M01AG02, M01AB03, M02AA21
Antihypertensive	C02, C03, C07, C08, C09
Antidiabetic	A10
Lipid-lowering	C10AA, C10BA, C10BX, C10AB, C10AC, C10AD, C10AX
Antiplatelet	B01AC, M03BC51, N02AB, N02BA01, N02BA51, N02BE01
Disease	ICD9 and ICD10
Liver disease	070, 570, 571, 572, 573
Hypertension	401–405, 362.11
Dyslipidemia	272
Ischemic heart disease	410–414, 429.2, 429.7
Alcohol intake	291, 303, 305.0, 357.5, 425.5, 535.3, 571.0, 571.1, 571.2, 571.3, 980.0
Heart failure	425, 428, 398.91, 402.01, 402.11, 402.91, 404.01, 404.03, 404.11, 404.13
Peripheral artery disease	442, 443, 440.1–440.4, 440.8, 440.9, 444.2, 444.8, 444.9, 447.1–447.6, 557.1, 557.9
Diabetes mellitus	250, 357.2, 362.0, 366.41

(*Continued*)

 © 2021. The Author(s).

Supplementary Table S1 (*Continued*)

Outcomes	ICD9 and 10
Chronic kidney disease	403, 404, 581, 582, 583, 585, 586, 587, 588, 589, 591, V56, 250.4, 271.4, 440.1, 442.1, 590.0, 590.2, 590.3, 590.8, 590.9, 593.0, 593.1, 593.2, 593.3, 593.4, 593.5, 593.7, 593.8, 593.9, 753.0, 753.1, 753.2, 753.6, 788.5, 274.10, 274.19, 446.21
Peptic ulcer disease	531, 532, 533, 534

Abbreviations: NOAC, non-vitamin K antagonist oral anticoagulant; NSAIDs, nonsteroidal anti-inflammatory drugs.

Supplementary Table S2 Use of OACs across the stroke severity in patients with AF

	OAC use, *n* (%)								
	Mild stroke *n* = 4,513 (42.1%)			Moderate stroke *n* = 2,582 (23.6%)			Severe stroke *n* = 3,861 (35.2%)		
	Early	Delayed	No use	Early	Delayed	No use	Early	Delayed	No use
Composite outcome analysis (*n* = 10,956)	1307 (29.0)	1,171 (25.9)	2,035 (45.1)	692 (26.8)	572 (22.2)	1,318 (51.0)	843 (21.8)	560 (14.5)	2,458 (63.7)

Abbreviation: OAC, oral anticoagulant.

 © 2021. The Author(s).

Supplementary Table S3 Use of antiplatelet treatment for strokes of varying severity in terms of the composite outcome before day-by-day matching

	Mild				Moderate				Severe			
	Use of anticoagulants		No use of anticoagulants		Use of anticoagulants		No use of anticoagulants		Use of anticoagulants		No use of anticoagulants	
Day	*N*	Use of antiplatelet	*N*	Use of antiplatelet	*N*	Use of antiplatelet	*N*	Use of antiplatelet	*N*	Use of antiplatelet	*N*	Use of antiplatelet
0	545	274 (50·28%)	3,968	3,239 (81·63%)	200	113 (56·5%)	2,382	1,957 (82·16%)	192	120 (62·5%)	3,669	3,131 (85·34%)
1	393	255 (64·89%)	3,545	2,963 (83·58%)	169	103 (60·95%)	2,176	1,830 (84·1%)	152	112 (73·68%)	3,452	2,981 (86·36%)
2	200	160 (80%)	3,324	2,791 (83·97%)	90	65 (72·22%)	2,070	1,751 (84·59%)	78	52 (66·67%)	3,310	2,887 (87·22%)
3	169	140 (82·84%)	3,138	2,640 (84·13%)	74	49 (66·22%)	1,974	1,687 (85·46%)	51	38 (74·51%)	3,163	2,775 (87·73%)
4	131	113 (86·26%)	2,993	2,517 (84·1%)	67	55 (82·09%)	1,883	1,613 (85·66%)	58	38 (65·52%)	3,042	2,691 (88·46%)
5	95	72 (75·79%)	2,885	2,434 (84·37%)	37	32 (86·49%)	1,833	1,571 (85·71%)	44	27 (61·36%)	2,938	2,614 (88·97%)
6	83	63 (75·9%)	2,785	2,364 (84·88%)	55	42 (76·36%)	1,769	1,522 (86·04%)	46	39 (84·78%)	2,850	2,540 (89·12%)
7	86	69 (80·23%)	2,678	2,277 (85·03%)	50	42 (84%)	1,702	1,464 (86·02%)	32	28 (87·5%)	2,788	2,485 (89·13%)
8	61	47 (77·05%)	2,605	2,224 (85·37%)	36	35 (97·22%)	1,652	1,420 (85·96%)	45	39 (86·67%)	2,708	2,420 (89·36%)
9	56	51 (91·07%)	2,521	2,154 (85·44%)	35	34 (97·14%)	1,605	1,379 (85·92%)	33	29 (87·88%)	2,644	2,366 (89·49%)
10	59	53 (89·83%)	2,429	2,078 (85·55%)	36	33 (91·67%)	1,562	1,341 (85·85%)	38	36 (94·74%)	2,575	2,303 (89·44%)
11	74	61 (82·43%)	2,330	2,002 (85·92%)	36	30 (83·33%)	1,512	1,302 (86·11%)	37	32 (86·49%)	2,513	2,251 (89·57%)
12	58	49 (84·48%)	2,245	1,932 (86·06%)	46	42 (91·3%)	1,447	1,243 (85·9%)	37	32 (86·49%)	2,451	2,199 (89·72%)
13	64	55 (85·94%)	2,157	1,861 (86·28%)	50	45 (90%)	1,382	1,189 (86·03%)	56	48 (85·71%)	2,378	2,136 (89·82%)
14	87	77 (88·51%)	2,046	1,768 (86·41%)	62	57 (91·94%)	1,306	1,121 (85·83%)	96	88 (91·67%)	2,251	2,025 (89·96%)
15	54	52 (96·3%)	1,975	1,704 (86·28%)	33	31 (93·94%)	1,252	1,071 (85·54%)	58	51 (87·93%)	2,164	1,947 (89·97%)
16	42	37 (88·1%)	1,920	1,661 (86·51%)	37	35 (94·59%)	1,196	1,020 (85·28%)	51	43 (84·31%)	2,097	1,894 (90·32%)
17	30	28 (93·33%)	1,870	1,618 (86·52%)	23	18 (78·26%)	1,158	995 (85·92%)	41	35 (85·37%)	2,037	1,842 (90·43%)
18	21	18 (85·71%)	1,825	1,585 (86·85%)	24	23 (95·83%)	1,120	963 (85·98%)	35	32 (91·43%)	1,975	1,783 (90·28%)
19	22	21 (95·45%)	1,789	1,551 (86·7%)	26	22 (84·62%)	1,079	930 (86·19%)	39	34 (87·18%)	1,919	1,735 (90·41%)
20	19	15 (78·95%)	1,763	1,534 (87·01%)	14	11 (78·57%)	1,048	902 (86·07%)	32	30 (93·75%)	1,872	1,691 (90·33%)
21	26	19 (73·08%)	1,723	1,507 (87·46%)	9	5 (55·56%)	1,029	888 (86·3%)	21	17 (80·95%)	1,830	1,657 (90·55%)
22	20	19 (95%)	1,695	1,486 (87·67%)	8	8 (100%)	1,013	873 (86·18%)	17	15 (88·24%)	1,799	1,633 (90·77%)
23	18	15 (83·33%)	1,670	1,469 (87·96%)	10	8 (80%)	996	860 (86·35%)	23	23 (100%)	1,759	1,595 (90·68%)
24	15	13 (86·67%)	1,646	1,448 (87·97%)	4	2 (50%)	979	850 (86·82%)	15	14 (93·33%)	1,730	1,568 (90·64%)
25	10	9 (90%)	1,630	1,434 (87·98%)	9	7 (77·78%)	958	832 (86·85%)	20	20 (100%)	1,686	1,527 (90·57%)
26	12	10 (83·33%)	1,610	1,421 (88·26%)	6	5 (83·33%)	946	823 (87%)	13	12 (92·31%)	1,660	1,503 (90·54%)
27	13	8 (61·54%)	1,590	1,409 (88·62%)	3	2 (66·67%)	933	813 (87·14%)	11	9 (81·82%)	1,628	1,475 (90·6%)
28	7	7 (100%)	1,570	1,394 (88·79%)	9	9 (100%)	914	799 (87·42%)	15	13 (86·67%)	1,598	1,452 (90·86%)
29	8	6 (75%)	1,551	1,381 (89·04%)	6	6 (100%)	900	789 (87·67%)	17	16 (94·12%)	1,554	1,411 (90·8%)

 © 2021. The Author(s).

Supplementary Table S4 Baseline characteristics of moderate stroke patients in terms of the composite outcome before and after 30 day-by-day matching

	Unmatched						Matched					
	Early treatment	No treatment	*p*-Value	Delayed treatment	No treatment	*p*-Value	Early treatment	No treatment	*p*-Value	Delayed treatment	No treatment	*p*-Value
Total	692 (100%)	1,318 (100%)		572 (100%)	1,318 (100%)		691 (100%)	691 (100%)		572 (100%)	572 (100%)	
Gender			0·7036			0·1293			0·6667			1
Female	354 (51·16%)	686 (52·05%)		276 (48·25%)	686 (52·05%)		354 (51·23%)	362 (52·39%)		276 (48·25%)	276 (48·25%)	
Male	338 (48·84%)	632 (47·95%)		296 (51·75%)	632 (47·95%)		337 (48·77%)	329 (47·61%)		296 (51·75%)	296 (51·75%)	
Age			**<0·0001**			**<0·0001**			0·8565			0·9883
≥80	274 (39·6%)	782 (59·33%)		232 (40·56%)	782 (59·33%)		274 (39·65%)	281 (40·67%)		232 (40·56%)	232 (40·56%)	
70–80	222 (32·08%)	347 (26·33%)		162 (28·32%)	347 (26·33%)		222 (32·13%)	224 (32·42%)		162 (28·32%)	160 (27·97%)	
<70	196 (28·32%)	189 (14·34%)		178 (31·12%)	189 (14·34%)		195 (28·22%)	186 (26·92%)		178 (31·12%)	180 (31·47%)	
Medication history												
Antihypertensive	610 (88·15%)	1,110 (84·22%)	**0·0171**	484 (84·62%)	1,110 (84·22%)	0·8274	609 (88·13%)	601 (86·98%)	0·5145	484 (84·62%)	489 (85·49%)	0·6784
Antidiabetic	187 (27·02%)	349 (26·48%)	0·7934	142 (24·83%)	349 (26·48%)	0·4512	187 (27·06%)	163 (23·59%)	0·1377	142 (24·83%)	134 (23·43%)	0·5804
Lipid-lowering agents	135 (19·51%)	224 (17%)	0·1622	110 (19·23%)	224 (17%)	0·2418	134 (19·39%)	130 (18·81%)	0·7843	110 (19·23%)	111 (19·41%)	0·9403
Anticoagulant	292 (42·2%)	152 (11·53%)	**<0·0001**	128 (22·38%)	152 (11·53%)	**<0·0001**	291 (42·11%)	308 (44·57%)	0·3561	128 (22·38%)	126 (22·03%)	0·8869
Antiplatelet	294 (42·49%)	743 (56·37%)	**<0·0001**	274 (47·9%)	743 (56·37%)	**0·0007**	294 (42·55%)	281 (40·67%)	0·478	274 (47·9%)	255 (44·58%)	0·2599
NSAID	692 (100%)	1,315 (99·77%)	1	571 (99·83%)	1,315 (99·77%)	0·8185	691 (100%)	691 (100%)	1	571 (99·83%)	572 (100%)	1
Medical history												
Liver disease	91 (13·15%)	178 (13·51%)	0·8242	73 (12·76%)	178 (13·51%)	0·6619	91 (13·17%)	85 (12·3%)	0·6283	73 (12·76%)	83 (14·51%)	0·3889
Peptic ulcer disease	192 (27·75%)	448 (33·99%)	**0·0043**	163 (28·5%)	448 (33·99%)	**0·019**	192 (27·79%)	189 (27·35%)	0·8567	163 (28·5%)	131 (22·9%)	**0·0304**
Hypertension	530 (76·59%)	1,080 (81·94%)	**0·0043**	432 (75·52%)	1,080 (81·94%)	**0·0014**	530 (76·7%)	530 (76·7%)	1	432 (75·52%)	442 (77·27%)	0·4863
Dyslipidemia	235 (33·96%)	369 (28%)	**0·0056**	197 (34·44%)	369 (28%)	**0·005**	235 (34·01%)	208 (30·1%)	0·1196	197 (34·44%)	195 (34·09%)	0·9008
IHD	287 (41·47%)	602 (45·68%)	0·0716	261 (45·63%)	602 (45·68%)	0·9853	287 (41·53%)	275 (39·8%)	0·5111	261 (45·63%)	264 (46·15%)	0·8587
ICH	11 (1·59%)	51 (3·87%)	**0·005**	14 (2·45%)	51 (3·87%)	0·1191	11 (1·59%)	11 (1·59%)	1	14 (2·45%)	14 (2·45%)	1
TIA	36 (5·2%)	74 (5·61%)	0·6994	31 (5·42%)	74 (5·61%)	0·865	36 (5·21%)	37 (5·35%)	0·9043	31 (5·42%)	25 (4·37%)	0·411
Alcohol intoxication	3 (0·43%)	14 (1·06%)	0·1436	7 (1·22%)	14 (1·06%)	0·7582	3 (0·43%)	1 (0·14%)	0·3166	7 (1·22%)	6 (1·05%)	0·7803
GI bleeding	78 (11·27%)	197 (14·95%)	**0·0227**	64 (11·19%)	197 (14·95%)	**0·0296**	78 (11·29%)	83 (12·01%)	0·675	64 (11·19%)	61 (10·66%)	0·7762
Hematuria	35 (5·06%)	75 (5·69%)	0·5535	28 (4·9%)	75 (5·69%)	0·4841	35 (5·07%)	32 (4·63%)	0·7071	28 (4·9%)	27 (4·72%)	0·8901
VTE	22 (3·18%)	26 (1·97%)	0·0923	8 (1·4%)	26 (1·97%)	0·3883	21 (3·04%)	21 (3·04%)	1	8 (1·4%)	7 (1·22%)	0·7949
Systemic embolism	9 (1·3%)	44 (3·34%)	**0·0067**	13 (2·27%)	44 (3·34%)	0·2133	9 (1·3%)	13 (1·88%)	0·39	13 (2·27%)	16 (2·8%)	0·5726
CHF	346 (50%)	642 (48·71%)	0·5826	246 (43·01%)	642 (48·71%)	**0·0225**	345 (49·93%)	350 (50·65%)	0·7879	246 (43·01%)	239 (41·78%)	0·6754
	30 (4·34%)	111 (8·42%)	**0·0007**	40 (6·99%)	111 (8·42%)	0·2926	30 (4·34%)	22 (3·18%)	0·2581	40 (6·99%)	33 (5·77%)	0·3971

 © 2021. The Author(s).

Supplementary Table S4 (*Continued*)

	Unmatched						Matched					
	Early treatment	No treatment	*p*-Value	Delayed treatment	No treatment	*p*-Value	Early treatment	No treatment	*p*-Value	Delayed treatment	No treatment	*p*-Value
Myocardial infarction												
PVD	54 (7·8%)	124 (9·41%)	0·2289	37 (6·47%)	124 (9·41%)	**0·0355**	54 (7·81%)	58 (8·39%)	0·6934	37 (6·47%)	37 (6·47%)	1
CVA	383 (55·35%)	691 (52·43%)	0·2126	306 (53·5%)	691 (52·43%)	0·669	382 (55·28%)	375 (54·27%)	0·7052	306 (53·5%)	313 (54·72%)	0·6779
Diabetes mellitus	236 (34·1%)	478 (36·27%)	0·3357	193 (33·74%)	478 (36·27%)	0·2918	236 (34·15%)	210 (30·39%)	0·1347	193 (33·74%)	191 (33·39%)	0·9004
CKD	190 (27·46%)	431 (32·7%)	**0·0156**	159 (27·8%)	431 (32·7%)	**0·0345**	190 (27·5%)	168 (24·31%)	0·1768	159 (27·8%)	150 (26·22%)	0·549
Secondary prevention												
Anticoagulant												
NOAC	168 (24·28%)	0		322 (56·29%)	0		168 (24·31%)	0		322 (56·29%)	0	
Warfarin	431 (62·28%)	0		227 (39·69%)	0		430 (62·23%)	0		227 (39·69%)	0	
Both	93 (13·44%)	0		23 (4·02%)	0		93 (13·46%)	0		23 (4·02%)	0	
Antiplatelet (AIS to index date)	Reference to Supplementary Table 3, moderate stroke, 0–6th day for early treatment and 7th–29th day for delayed treatment						459 (66·43%)	456 (65·99%)	0·8645	510 (89·16%)	524 (91·61%)	0·1603

Abbreviations: AIS, acute ischemic stroke; CHF, congestive heart failure; CKD, chronic kidney disease; CVA, cerebrovascular accident; GI, gastrointestinal; ICH, intracranial hemorrhage; IHD, ischemic heart disease; NOAC, non-vitamin K antagonist oral anticoagulant; NSAIDs, nonsteroidal anti-inflammatory drugs; PVD, peripheral vascular disease; TIA, transient ischemic attack; VTE, venous thromboembolism.
Note: Data are mean $\pm$ standard deviation (SD) or *n* (%).

 © 2021. The Author(s).

Supplementary Table S5 Baseline characteristics of severe stroke patients in terms of the composite outcome before and after 30 day-by-day matching

	Unmatched						Matched					
	Early treatment	No treatment	*p*-Value	Delayed treatment	No treatment	*p*-Value	Early treatment	No treatment	*p*-Value	Delayed treatment	No treatment	*p*-Value
Total	843 (100%)	2,458 (100%)		560 (100%)	2,458 (100%)		843 (100%)	843 (100%)		560 (100%)	560 (100%)	
Gender			0·9262			0·7881			0·6558			0·8086
Female	493 (58·48%)	1,433 (58·3%)		323 (57·68%)	1,433 (58·3%)		493 (58·48%)	502 (59·55%)		323 (57·68%)	327 (58·39%)	
Male	350 (41·52%)	1,025 (41·7%)		237 (42·32%)	1,025 (41·7%)		350 (41·52%)	341 (40·45%)		237 (42·32%)	233 (41·61%)	
Age			<0·0001			<0·0001			0·5773			0·6963
≥80	344 (40·81%)	1,277 (51·95%)		229 (40·89%)	1,277 (51·95%)		344 (40·81%)	323 (38·32%)		229 (40·89%)	233 (41·61%)	
70–80	288 (34·16%)	767 (31·2%)		187 (33·39%)	767 (31·2%)		288 (34·16%)	299 (35·47%)		187 (33·39%)	195 (34·82%)	
<70	211 (25·03%)	414 (16·84%)		144 (25·71%)	414 (16·84%)		211 (25·03%)	221 (26·22%)		144 (25·71%)	132 (23·57%)	
Medication history												
Antihypertensive	745 (88·37%)	2,133 (86·78%)	0·2313	480 (85·71%)	2,133 (86·78%)	0·5052	745 (88·37%)	749 (88·85%)	0·7591	480 (85·71%)	481 (85·89%)	0·9318
Antidiabetic	242 (28·71%)	683 (27·79%)	0·6077	125 (22·32%)	683 (27·79%)	0·0084	242 (28·71%)	247 (29·3%)	0·7884	125 (22·32%)	123 (21·96%)	0·8856
Lipid-lowering agents	176 (20·88%)	453 (18·43%)	0·1183	128 (22·86%)	453 (18·43%)	0·0165	176 (20·88%)	179 (21·23%)	0·8578	128 (22·86%)	123 (21·96%)	0·7201
Anticoagulant	333 (39·5%)	352 (14·32%)	**<0·0001**	119 (21·25%)	352 (14·32%)	<0·0001	333 (39·5%)	311 (36·89%)	0·2701	119 (21·25%)	131 (23·39%)	0·3892
Antiplatelet	373 (44·25%)	1,355 (55·13%)	**<0·0001**	234 (41·79%)	1,355 (55·13%)	<0·0001	373 (44·25%)	376 (44·6%)	0·8831	234 (41·79%)	221 (39·46%)	0·429
NSAIDs	841 (99·76%)	2,446 (99·51%)	0·3333	559 (99·82%)	2,446 (99·51%)	0·3126	841 (99·76%)	841 (99·76%)	1	559 (99·82%)	560 (100%)	1
Medical history												
Liver disease	105 (12·46%)	275 (11·19%)	0·3197	64 (11·43%)	275 (11·19%)	0·8707	105 (12·46%)	85 (10·08%)	0·1235	64 (11·43%)	65 (11·61%)	0·9254
Peptic ulcer disease	239 (28·35%)	761 (30·96%)	0·1549	134 (23·93%)	761 (30·96%)	0·001	239 (28·35%)	231 (27·4%)	0·6639	134 (23·93%)	127 (22·68%)	0·6208
Hypertension	653 (77·46%)	2,002 (81·45%)	0·0118	433 (77·32%)	2,002 (81·45%)	0·0256	653 (77·46%)	657 (77·94%)	0·815	433 (77·32%)	432 (77·14%)	0·9432
Dyslipidemia	272 (32·27%)	724 (29·45%)	0·125	188 (33·57%)	724 (29·45%)	0·0556	272 (32·27%)	262 (31·08%)	0·6006	188 (33·57%)	194 (34·64%)	0·7053
IHD	340 (40·33%)	1,138 (46·3%)	0·0026	220 (39·29%)	1,138 (46·3%)	0·0026	340 (40·33%)	317 (37·6%)	0·2507	220 (39·29%)	213 (38·04%)	0·6675
ICH	21 (2·49%)	64 (2·6%)	0·8586	12 (2·14%)	64 (2·6%)	0·5299	21 (2·49%)	26 (3·08%)	0·4595	12 (2·14%)	16 (2·86%)	0·4439
TIA	44 (5·22%)	111 (4·52%)	0·4047	19 (3·39%)	111 (4·52%)	0·2375	44 (5·22%)	37 (4·39%)	0·4254	19 (3·39%)	16 (2·86%)	0·6064
Alcohol intoxication	6 (0·71%)	21 (0·85%)	0·6916	6 (1·07%)	21 (0·85%)	0·6225	6 (0·71%)	6 (0·71%)	1	6 (1·07%)	5 (0·89%)	0·7619
GI bleeding	74 (8·78%)	283 (11·51%)	0·0273	48 (8·57%)	283 (11·51%)	0·0444	74 (8·78%)	68 (8·07%)	0·5988	48 (8·57%)	38 (6·79%)	0·2617
Hematuria	34 (4·03%)	84 (3·42%)	0·406	23 (4·11%)	84 (3·42%)	0·4257	34 (4·03%)	32 (3·8%)	0·8017	23 (4·11%)	23 (4·11%)	1
VTE	25 (2·97%)	52 (2·12%)	0·1582	10 (1·79%)	52 (2·12%)	0·6195	25 (2·97%)	20 (2·37%)	0·4499	10 (1·79%)	7 (1·25%)	0·4634
Systemic embolism	25 (2·97%)	86 (3·5%)	0·4587	12 (2·14%)	86 (3·5%)	0·1023	25 (2·97%)	19 (2·25%)	0·3594	12 (2·14%)	13 (2·32%)	0·8397
CHF	442 (52·43%)	1,210 (49·23%)	0·1083	250 (44·64%)	1,210 (49·23%)	0·0501	442 (52·43%)	432 (51·25%)	0·626	250 (44·64%)	254 (45·36%)	0·8101
Myocardial infarction	61 (7·24%)	197 (8·01%)	0·4674	29 (5·18%)	197 (8·01%)	0·0214	61 (7·24%)	62 (7·35%)	0·9254	29 (5·18%)	26 (4·64%)	0·6783
PVD	72 (8·54%)	253 (10·29%)	0·1407	35 (6·25%)	253 (10·29%)	0·0033	72 (8·54%)	69 (8·19%)	0·7918	35 (6·25%)	44 (7·86%)	0·2936
CVA	490 (58·13%)	1,438 (58·5%)	0·848	379 (67·68%)	1,438 (58·5%)	<0·0001	490 (58·13%)	491 (58·24%)	0·9606	379 (67·68%)	386 (68·93%)	0·653

 © 2021. The Author(s).

Supplementary Table S5 (*Continued*)

		Unmatched					Matched					
	Early treatment	No treatment	*p*-Value	Delayed treatment	No treatment	*p*-Value	Early treatment	No treatment	*p*-Value	Delayed treatment	No treatment	*p*-Value
Diabetes mellitus	293 (34·76%)	893 (36·33%)	0·4113	173 (30·89%)	893 (36·33%)	0·0151	293 (34·76%)	291 (34·52%)	0·9185	173 (30·89%)	161 (28·75%)	0·4332
CKD	221 (26·22%)	736 (29·94%)	0·0396	133 (23·75%)	736 (29·94%)	0·0035	221 (26·22%)	221 (26·22%)	1	133 (23·75%)	132 (23·57%)	0·9439
Secondary prevention												
Anticoagulant												
NOAC	166 (19·69%)	0		312 (55·71%)	0		166 (19·69%)	0		312 (55·71%)	0	
Warfarin	580 (68·8%)	0		235 (41·96%)	0		580 (68·8%)	0		235 (41·96%)	0	
Both	97 (11·51%)	0		13 (2·32%)	0		97 (11·51%)	0		13 (2·32%)	0	
Antiplatelet (AIS to index date)	Reference to Supplementary Table 3, severe stroke, 0–12th day for early treatment and 13th–29th day for delayed treatment						622 (73·78%)	619 (73·43%)	0·8683	500 (89·29%)	502 (89·64%)	0·8457

Abbreviations: AIS, acute ischemic stroke; CHF, congestive heart failure; CKD, chronic kidney disease; CVA, cerebrovascular accident; GI, gastrointestinal; ICH, intracranial hemorrhage; IHD, ischemic heart disease; NOAC, non-vitamin K antagonist oral anticoagulant; NSAIDs, nonsteroidal anti-inflammatory drugs; PVD, peripheral vascular disease; TIA, transient ischemic attack; VTE, venous thromboembolism.
Note: Data are mean ± standard deviation (SD) or *n* (%).

 © 2021. The Author(s).

Supplementary Table S6 Baseline characteristics of mild stroke patients in terms of the effectiveness outcome before and after 30 day-by-day matching

	Unmatched						Matched					
	Early treatment	No treatment	*p*-Value	Delayed treatment	No treatment	*p*-Value	Early treatment	No treatment	*p*-Value	Delayed treatment	No treatment	*p*-Value
Total	1,325 (100%)	2,091 (100%)		1,198 (100%)	2,091 (100%)		1,325 (100%)	1,325 (100%)		1,198 (100%)	1,198 (100%)	
Gender			0·4496			0·132			0·171			0·8034
Female	603 (45·51%)	924 (44·19%)		497 (41·49%)	924 (44·19%)		603 (45·51%)	568 (42·87%)		497 (41·49%)	491 (40·98%)	
Male	722 (54·49%)	1167 (55·81%)		701 (58·51%)	1,167 (55·81%)		722 (54·49%)	757 (57·13%)		701 (58·51%)	707 (59·02%)	
Age			<0·0001			<0·0001			0·8893			0·439
≥80	441 (33·28%)	949 (45·38%)		396 (33·06%)	949 (45·38%)		441 (33·28%)	435 (32·83%)		396 (33·06%)	421 (35·14%)	
70–80	477 (36%)	673 (32·19%)		435 (36·31%)	673 (32·19%)		477 (36%)	489 (36·91%)		435 (36·31%)	435 (36·31%)	
< 70	407 (30·72%)	469 (22·43%)		367 (30·63%)	469 (22·43%)		407 (30·72%)	401 (30·26%)		367 (30·63%)	342 (28·55%)	
Medication history												
Antihypertensive	1,175 (88·68%)	1,818 (86·94%)	0·1335	1,015 (84·72%)	1,818 (86·94%)	0·0763	1,175 (88·68%)	1,174 (88·6%)	0·9512	1,015 (84·72%)	1,004 (83·81%)	0·5371
Antidiabetic	372 (28·08%)	614 (29·36%)	0·4181	306 (25·54%)	614 (29·36%)	0·0188	372 (28·08%)	362 (27·32%)	0·6642	306 (25·54%)	293 (24·46%)	0·5397
Lipid-lowering agents	313 (23·62%)	433 (20·71%)	0·0445	285 (23·79%)	433 (20·71%)	0·0395	313 (23·62%)	281 (21·21%)	0·1361	285 (23·79%)	291 (24·29%)	0·7742
Anticoagulant	636 (48%)	242 (11·57%)	<0·0001	280 (23·37%)	242 (11·57%)	<0·0001	636 (48%)	633 (47·77%)	0·9071	280 (23·37%)	274 (22·87%)	0·7713
Antiplatelet	606 (45·74%)	1,227 (58·68%)	<0·0001	638 (53·26%)	1,227 (58·68%)	0·0025	606 (45·74%)	616 (46·49%)	0·6968	638 (53·26%)	636 (53·09%)	0·9347
NSAIDs	1,325 (100%)	2,088 (99·86%)	1	1,198 (100%)	2,088 (99·86%)	1	1,325 (100%)	1,325 (100%)	0·3177	1,198 (100%)	1,198 (100%)	0·3177
Medical history												
Liver disease	181 (13·66%)	313 (14·97%)	0·2893	196 (16·36%)	313 (14·97%)	0·2883	181 (13·66%)	174 (13·13%)	0·6897	196 (16·36%)	161 (13·44%)	0·0446
Peptic ulcer disease	362 (27·32%)	732 (35·01%)	<0·0001	354 (29·55%)	732 (35·01%)	0·0014	362 (27·32%)	361 (27·25%)	0·9652	354 (29·55%)	317 (26·46%)	0·0923
Hypertension	1,041 (78·57%)	1,743 (83·36%)	0·0004	946 (78·96%)	1,743 (83·36%)	0·0017	1,041 (78·57%)	1,057 (79·77%)	0·4441	946 (78·96%)	960 (80·13%)	0·4783
Dyslipidemia	461 (34·79%)	724 (34·62%)	0·92	426 (35·56%)	724 (34·62%)	0·5886	461 (34·79%)	452 (34·11%)	0·7129	426 (35·56%)	409 (34·14%)	0·4661
IHD	626 (47·25%)	1,055 (50·45%)	0·0675	557 (46·49%)	1,055 (50·45%)	0·0288	626 (47·25%)	625 (47·17%)	0·969	557 (46·49%)	544 (45·41%)	0·5941
ICH	26 (1·96%)	65 (3·11%)	0·0426	21 (1·75%)	65 (3·11%)	0·0191	26 (1·96%)	27 (2·04%)	0·8896	21 (1·75%)	21 (1·75%)	1
TIA	97 (7·32%)	163 (7·8%)	0·6103	76 (6·34%)	163 (7·8%)	0·1228	97 (7·32%)	93 (7·02%)	0·7633	76 (6·34%)	69 (5·76%)	0·5487
Alcohol intoxication	10 (0·75%)	25 (1·2%)	0·2125	10 (0·83%)	25 (1·2%)	0·3317	10 (0·75%)	16 (1·21%)	0·237	10 (0·83%)	6 (0·5%)	0·3157
GI bleeding	129 (9·74%)	304 (14·54%)	<0·0001	123 (10·27%)	304 (14·54%)	0·0005	129 (9·74%)	124 (9·36%)	0·741	123 (10·27%)	109 (9·1%)	0·3335
Hematuria	65 (4·91%)	95 (4·54%)	0·6252	62 (5·18%)	95 (4·54%)	0·4133	65 (4·91%)	69 (5·21%)	0·7229	62 (5·18%)	65 (5·43%)	0·7844
VTE	27 (2·04%)	33 (1·58%)	0·3191	18 (1·5%)	33 (1·58%)	0·8657	27 (2·04%)	27 (2·04%)	1	18 (1·5%)	14 (1·17%)	0·4765
Systemic embolism	29 (2·19%)	66 (3·16%)	0·0937	29 (2·42%)	66 (3·16%)	0·2254	29 (2·19%)	30 (2·26%)	0·8953	29 (2·42%)	27 (2·25%)	0·7868
CHF	612 (46·19%)	943 (45·1%)	0·5328	459 (38·31%)	943 (45·1%)	0·0002	612 (46·19%)	607 (45·81%)	0·8455	459 (38·31%)	470 (39·23%)	0·6446
Myocardial infarction	90 (6·79%)	198 (9·47%)	0·0061	70 (5·84%)	198 (9·47%)	0·0003	90 (6·79%)	81 (6·11%)	0·4767	70 (5·84%)	63 (5·26%)	0·5323
PVD	108 (8·15%)	213 (10·19%)	0·0469	79 (6·59%)	213 (10·19%)	0·0005	108 (8·15%)	112 (8·45%)	0·7782	79 (6·59%)	66 (5·51%)	0·2654
CVA	616 (46·49%)	977 (46·72%)	0·894	567 (47·33%)	977 (46·72%)	0·738	616 (46·49%)	610 (46·04%)	0·8152	567 (47·33%)	559 (46·66%)	0·7433
Diabetes mellitus	466 (35·17%)	821 (39·26%)	0·0161	401 (33·47%)	821 (39·26%)	0·0009	466 (35·17%)	474 (35·77%)	0·7453	401 (33·47%)	374 (31·22%)	0·2383
CKD	368 (27·77%)	701 (33·52%)	0·0004	310 (25·88%)	701 (33·52%)	<0·0001	368 (27·77%)	378 (28·53%)	0·6658	310 (25·88%)	299 (24·96%)	0·6058

 © 2021. The Author(s).

Supplementary Table S6 (*Continued*)

	Unmatched						Matched					
	Early treatment	No treatment	*p*-Value	Delayed treatment	No treatment	*p*-Value	Early treatment	No treatment	*p*-Value	Delayed treatment	No treatment	*p*-Value
Secondary prevention												
Anticoagulant												
NOAC	361 (27·25%)	0		680 (56·76%)	0		361 (27·25%)	0		680 (56·76%)	0	
Warfarin	821 (61·96%)	0		459 (38·31%)	0		821 (61·96%)	0		459 (38·31%)	0	
Both	143 (10·79%)	0		59 (4·92%)	0		143 (10·79%)	0		59 (4·92%)	0	
Antiplatelet (AIS to index date)	Reference to Supplementary Table 7, mild stroke, 0–3rd day for early treatment and 4th–29th day for delayed treatment						838 (63·25%)	830 (62·64%)	0·7476	1,009 (84·22%)	1,000 (83·47%)	0·6173

Abbreviations: AIS, acute ischemic stroke; CHF, congestive heart failure; CKD, chronic kidney disease; CVA, cerebrovascular accident; GI, gastrointestinal; ICH, intracranial hemorrhage; IHD, ischemic heart disease; NOAC, non-vitamin K antagonist oral anticoagulant; NSAIDs, nonsteroidal anti-inflammatory drugs; PVD, peripheral vascular disease; TIA, transient ischemic attack; VTE, venous thromboembolism.
Note: Data are mean $\pm$ standard deviation (SD) or *n* (%).

 © 2021. The Author(s).

Supplementary Table S7 Use of antiplatelet treatment for strokes of varying severity in terms of the effectiveness outcome before day-by-day matching

	Mild				Moderate				Severe			
	Use of anticoagulants		No use of anticoagulants		Use of anticoagulants		No use of anticoagulants		Use of anticoagulants		No use of anticoagulants	
Day	*N*	Use of antiplatelet	*N*	Use of antiplatelet	*N*	Use of antiplatelet	*N*	Use of antiplatelet	*N*	Use of antiplatelet	*N*	Use of antiplatelet
0	554	279 (50·36%)	4,060	3,309 (81·5%)	205	114 (55·61%)	2,514	2,055 (81·74%)	206	130 (63·11%)	3,990	3,417 (85·64%)
1	400	258 (64·5%)	3,630	3,030 (83·47%)	179	111 (62·01%)	2,298	1,920 (83·55%)	157	115 (73·25%)	3,765	3,263 (86·67%)
2	201	161 (80·1%)	3,408	2,856 (83·8%)	95	69 (72·63%)	2,183	1,835 (84·06%)	83	56 (67·47%)	3,613	3,162 (87·52%)
3	170	140 (82·35%)	3,220	2,704 (83·98%)	76	51 (67·11%)	2,084	1,768 (84·84%)	54	40 (74·07%)	3,456	3,043 (88·05%)
4	135	116 (85·93%)	3,071	2,578 (83·95%)	71	58 (81·69%)	1,988	1,690 (85·01%)	63	43 (68·25%)	3,324	2,949 (88·72%)
5	96	73 (76·04%)	2,964	2,496 (84·21%)	39	34 (87·18%)	1,936	1,646 (85·02%)	46	28 (60·87%)	3,217	2,870 (89·21%)
6	83	63 (75·9%)	2,866	2,427 (84·68%)	58	44 (75·86%)	1,869	1,595 (85·34%)	51	44 (86·27%)	3,123	2,790 (89·34%)
7	87	70 (80·46%)	2,758	2,339 (84·81%)	52	44 (84·62%)	1,800	1,535 (85·28%)	36	32 (88·89%)	3,056	2,730 (89·33%)
8	61	47 (77·05%)	2,686	2,287 (85·15%)	36	35 (97·22%)	1,754	1,493 (85·12%)	48	42 (87·5%)	2,974	2,662 (89·51%)
9	58	52 (89·66%)	2,601	2,217 (85·24%)	35	34 (97·14%)	1,707	1,453 (85·12%)	38	33 (86·84%)	2,904	2,603 (89·63%)
10	61	54 (88·52%)	2,508	2,141 (85·37%)	36	33 (91·67%)	1,663	1,414 (85·03%)	39	37 (94·87%)	2,835	2,540 (89·59%)
11	74	61 (82·43%)	2,412	2,068 (85·74%)	40	34 (85%)	1,611	1,372 (85·16%)	38	33 (86·84%)	2,772	2,487 (89·72%)
12	60	51 (85%)	2,334	2,005 (85·9%)	50	46 (92%)	1,545	1,313 (84·98%)	40	35 (87·5%)	2,704	2,429 (89·83%)
13	64	55 (85·94%)	2,251	1,940 (86·18%)	53	46 (86·79%)	1,478	1,258 (85·12%)	64	56 (87·5%)	2,625	2,359 (89·87%)
14	90	80 (88·89%)	2,141	1,848 (86·31%)	63	58 (92·06%)	1,400	1,189 (84·93%)	106	98 (92·45%)	2,488	2,238 (89·95%)
15	57	55 (96·49%)	2,068	1,782 (86·17%)	35	33 (94·29%)	1,348	1,141 (84·64%)	61	52 (85·25%)	2,399	2,160 (90·04%)
16	42	37 (88·1%)	2,015	1,741 (86·4%)	38	35 (92·11%)	1,290	1,090 (84·5%)	56	48 (85·71%)	2,328	2,102 (90·29%)
17	32	30 (93·75%)	1,967	1,699 (86·38%)	23	18 (78·26%)	1,254	1,067 (85·09%)	45	39 (86·67%)	2,265	2,048 (90·42%)
18	23	19 (82·61%)	1,923	1,666 (86·64%)	25	24 (96%)	1,216	1,035 (85·12%)	36	33 (91·67%)	2,204	1,990 (90·29%)
19	22	21 (95·45%)	1,890	1,635 (86·51%)	27	23 (85·19%)	1,175	1002 (85·28%)	41	36 (87·8%)	2,146	1,940 (90·4%)
20	19	15 (78·95%)	1,864	1,618 (86·8%)	15	12 (80%)	1,146	976 (85·17%)	33	31 (93·94%)	2,098	1,895 (90·32%)
21	26	19 (73·08%)	1,826	1,594 (87·29%)	9	5 (55·56%)	1,127	962 (85·36%)	23	19 (82·61%)	2,053	1,858 (90·5%)
22	20	19 (95%)	1,800	1,573 (87·39%)	10	9 (90%)	1,112	949 (85·34%)	17	15 (88·24%)	2,020	1,832 (90·69%)
23	19	16 (84·21%)	1,774	1,555 (87·66%)	10	8 (80%)	1,093	934 (85·45%)	24	24 (100%)	1,981	1,796 (90·66%)
24	17	14 (82·35%)	1,751	1,535 (87·66%)	8	5 (62·5%)	1,075	923 (85·86%)	16	15 (93·75%)	1,950	1,767 (90·62%)
25	12	11 (91·67%)	1,733	1,519 (87·65%)	9	7 (77·78%)	1,056	907 (85·89%)	23	22 (95·65%)	1,904	1,725 (90·6%)
26	12	10 (83·33%)	1,715	1,507 (87·87%)	6	5 (83·33%)	1,043	897 (86%)	15	14 (93·33%)	1,872	1,695 (90·54%)
27	13	8 (61·54%)	1,698	1,498 (88·22%)	3	2 (66·67%)	1,033	889 (86·06%)	11	9 (81·82%)	1,841	1,668 (90·6%)
28	7	7 (100%)	1,680	1,484 (88·33%)	12	12 (100%)	1,010	872 (86·34%)	15	13 (86·67%)	1,811	1,644 (90·78%)
29	8	6 (75%)	1,662	1,471 (88·51%)	8	7 (87·5%)	998	864 (86·57%)	21	19 (90·48%)	1,766	1,602 (90·71%)

 © 2021. The Author(s).

Supplementary Table S8 Baseline characteristics of moderate stroke patients in terms of the effectiveness outcome before and after 30 day-by-day matching

	Unmatched						Matched					
	Early treatment	No treatment	*p*-Value	Delayed treatment	No treatment	*p*-Value	Early treatment	No treatment	*p*-Value	Delayed treatment	No treatment	*p*-Value
Total	723 (100%)	1,393 (100%)		603 (100%)	1,393 (100%)		723 (100%)	723 (100%)		602 (100%)	602 (100%)	
Gender			0·6826			0·0454			0·6358			0·954
Female	369 (51·04%)	724 (51·97%)		284 (47·1%)	724 (51·97%)		369 (51·04%)	378 (52·28%)		284 (47·18%)	283 (47·01%)	
Male	354 (48·96%)	669 (48·03%)		319 (52·9%)	669 (48·03%)		354 (48·96%)	345 (47·72%)		318 (52·82%)	319 (52·99%)	
Age			<0·0001			<0·0001			0·5613			0·918
≥80	290 (40·11%)	829 (59·51%)		249 (41·29%)	829 (59·51%)		290 (40·11%)	310 (42·88%)		248 (41·2%)	254 (42·19%)	
70–80	233 (32·23%)	365 (26·2%)		170 (28·19%)	365 (26·2%)		233 (32·23%)	224 (30·98%)		170 (28·24%)	170 (28·24%)	
<70	200 (27·66%)	199 (14·29%)		184 (30·51%)	199 (14·29%)		200 (27·66%)	189 (26·14%)		184 (30·56%)	178 (29·57%)	
Medication history												
Antihypertensive	634 (87·69%)	1,174 (84·28%)	0·0348	510 (84·58%)	1,174 (84·28%)	0·8661	634 (87·69%)	628 (86·86%)	0·6359	509 (84·55%)	510 (84·72%)	0·9363
Antidiabetic	198 (27·39%)	374 (26·85%)	0·7918	143 (23·71%)	374 (26·85%)	0·1423	198 (27·39%)	192 (26·56%)	0·7222	143 (23·75%)	145 (24·09%)	0·8925
Lipid-lowering agents	140 (19·36%)	236 (16·94%)	0·1669	117 (19·4%)	236 (16·94%)	0·1858	140 (19·36%)	132 (18·26%)	0·5903	116 (19·27%)	115 (19·1%)	0·9417
Anticoagulant	304 (42·05%)	161 (11·56%)	<0·0001	137 (22·72%)	161 (11·56%)	<0·0001	304 (42·05%)	318 (43·98%)	0·4571	136 (22·59%)	125 (20·76%)	0·4417
Antiplatelet	307 (42·46%)	790 (56·71%)	<0·0001	289 (47·93%)	790 (56·71%)	0·0003	307 (42·46%)	306 (42·32%)	0·9576	289 (48·01%)	288 (47·84%)	0·954
NSAIDs	723 (100%)	1,388 (99·64%)	1	602 (99·83%)	1,388 (99·64%)	0·4693	723 (100%)	723 (100%)	0·3177	601 (99·83%)	601 (99·83%)	1
Medical history												
Liver disease	94 (13%)	189 (13·57%)	0·7166	76 (12·6%)	189 (13·57%)	0·5599	94 (13%)	107 (14·8%)	0·3231	76 (12·62%)	75 (12·46%)	0·9307
Peptic ulcer disease	197 (27·25%)	471 (33·81%)	0·0021	171 (28·36%)	471 (33·81%)	0·0166	197 (27·25%)	174 (24·07%)	0·1661	170 (28·24%)	140 (23·26%)	0·048
Hypertension	552 (76·35%)	1,142 (81·98%)	0·0021	454 (75·29%)	1,142 (81·98%)	0·0006	552 (76·35%)	553 (76·49%)	0·9506	453 (75·25%)	468 (77·74%)	0·308
Dyslipidemia	239 (33·06%)	389 (27·93%)	0·0143	204 (33·83%)	389 (27·93%)	0·008	239 (33·06%)	230 (31·81%)	0·6131	204 (33·89%)	194 (32·23%)	0·5401
IHD	299 (41·36%)	635 (45·59%)	0·0631	276 (45·77%)	635 (45·59%)	0·9389	299 (41·36%)	303 (41·91%)	0·831	275 (45·68%)	273 (45·35%)	0·9079
ICH	12 (1·66%)	53 (3·8%)	0·0067	14 (2·32%)	53 (3·8%)	0·0912	12 (1·66%)	13 (1·8%)	0·8401	14 (2·33%)	9 (1·5%)	0·2925
TIA	37 (5·12%)	78 (5·6%)	0·6429	31 (5·14%)	78 (5·6%)	0·6789	37 (5·12%)	31 (4·29%)	0·4561	31 (5·15%)	32 (5·32%)	0·897
Alcohol intoxication	4 (0·55%)	14 (1·01%)	0·2832	8 (1·33%)	14 (1·01%)	0·5274	4 (0·55%)	4 (0·55%)	1	8 (1·33%)	9 (1·5%)	0·807
GI bleeding	85 (11·76%)	222 (15·94%)	0·0096	69 (11·44%)	222 (15·94%)	0·009	85 (11·76%)	66 (9·13%)	0·1023	69 (11·46%)	52 (8·64%)	0·1032
Hematuria	39 (5·39%)	78 (5·6%)	0·8447	32 (5·31%)	78 (5·6%)	0·7925	39 (5·39%)	41 (5·67%)	0·818	32 (5·32%)	32 (5·32%)	1
VTE	22 (3·04%)	29 (2·08%)	0·1716	9 (1·49%)	29 (2·08%)	0·3764	22 (3·04%)	22 (3·04%)	1	9 (1·5%)	7 (1·16%)	0·6147
Systemic embolism	10 (1·38%)	48 (3·45%)	0·0058	13 (2·16%)	48 (3·45%)	0·1242	10 (1·38%)	9 (1·24%)	0·8174	12 (1·99%)	7 (1·16%)	0·2476
CHF	359 (49·65%)	686 (49·25%)	0·8587	258 (42·79%)	686 (49·25%)	0·0079	359 (49·65%)	380 (52·56%)	0·2693	257 (42·69%)	251 (41·69%)	0·7262
Myocardial infarction	32 (4·43%)	119 (8·54%)	0·0005	41 (6·8%)	119 (8·54%)	0·1878	32 (4·43%)	26 (3·6%)	0·4213	41 (6·81%)	45 (7·48%)	0·6544
PVD	57 (7·88%)	132 (9·48%)	0·2233	38 (6·3%)	132 (9·48%)	0·0197	57 (7·88%)	51 (7·05%)	0·5484	37 (6·15%)	31 (5·15%)	0·4538
CVA	396 (54·77%)	724 (51·97%)	0·2214	323 (53·57%)	724 (51·97%)	0·5133	396 (54·77%)	376 (52·01%)	0·2917	322 (53·49%)	316 (52·49%)	0·729
Diabetes mellitus	249 (34·44%)	509 (36·54%)	0·3393	196 (32·5%)	509 (36·54%)	0·0833	249 (34·44%)	234 (32·37%)	0·403	196 (32·56%)	201 (33·39%)	0·7592
CKD	199 (27·52%)	458 (32·88%)	0·0116	168 (27·86%)	458 (32·88%)	0·0265	199 (27·52%)	184 (25·45%)	0·3714	168 (27·91%)	164 (27·24%)	0·7964

(Continued)

 © 2021. The Author(s).

Supplementary Table S8 (*Continued*)

	Unmatched						Matched					
	Early treatment	No treatment	*p*-Value	Delayed treatment	No treatment	*p*-Value	Early treatment	No treatment	*p*-Value	Delayed treatment	No treatment	*p*-Value
Secondary prevention												
Anticoagulant												
NOAC	173 (23·93%)	0		345 (57·21%)	0		173 (23·93%)	0		345 (57·31%)	0	
Warfarin	453 (62·66%)	0		235 (38·97%)	0		453 (62·66%)	0		234 (38·87%)	0	
Both	97 (13·42%)	0		23 (3·81%)	0		97 (13·42%)	0		23 (3·82%)	0	
Antiplatelet (AIS to index date)	Reference to Supplementary Table 7, moderate stroke, 0–6th day for early treatment and 7th–29th day for delayed treatment						482 (66·67%)	475 (65·7%)	0·6972	535 (88·87%)	549 (91·2%)	0·178

Abbreviations: AIS, acute ischemic stroke; CHF, congestive heart failure; CKD, chronic kidney disease; CVA, cerebrovascular accident; GI, gastrointestinal; ICH, intracranial hemorrhage; IHD, ischemic heart disease; NOAC, non-vitamin K antagonist oral anticoagulant; NSAIDs, nonsteroidal anti-inflammatory drugs; PVD, peripheral vascular disease; TIA, transient ischemic attack; VTE, venous thromboembolism.
Note: Data are mean ± standard deviation (SD) or *n* (%).

 © 2021. The Author(s).

Supplementary Table S9 Baseline characteristics of severe stroke patients in terms of the effectiveness outcome before and after 30 day-by-day matching

	Unmatched						Matched					
	Early treatment	No treatment	*p*-Value	Delayed treatment	No treatment	*p*-Value	Early treatment	No treatment	*p*-Value	Delayed treatment	No treatment	*p*-Value
Total	899 (100%)	2,690 (100%)		607 (100%)	2,690 (100%)		899 (100%)	899 (100%)		607 (100%)	607 (100%)	
Gender			0·9215			0·478			0·3885			0·7288
Female	519 (57·73%)	1,558 (57·92%)		342 (56·34%)	1,558 (57·92%)		519 (57·73%)	537 (59·73%)		342 (56·34%)	336 (55·35%)	
Male	380 (42·27%)	1,132 (42·08%)		265 (43·66%)	1,132 (42·08%)		380 (42·27%)	362 (40·27%)		265 (43·66%)	271 (44·65%)	
Age			<0·0001			<0·0001			0·7902			0·4881
≥80	365 (40·6%)	1,408 (52·34%)		247 (40·69%)	1,408 (52·34%)		365 (40·6%)	352 (39·15%)		247 (40·69%)	267 (43·99%)	
70–80	303 (33·7%)	826 (30·71%)		203 (33·44%)	826 (30·71%)		303 (33·7%)	315 (35·04%)		203 (33·44%)	188 (30·97%)	
<70	231 (25·7%)	456 (16·95%)		157 (25·86%)	456 (16·95%)		231 (25·7%)	232 (25·81%)		157 (25·86%)	152 (25·04%)	
Medication history												
Antihypertensive	795 (88·43%)	2,332 (86·69%)	0·1774	513 (84·51%)	2,332 (86·69%)	0·1589	795 (88·43%)	799 (88·88%)	0·7661	513 (84·51%)	514 (84·68%)	0·9366
Antidiabetic	252 (28·03%)	745 (27·7%)	0·8456	135 (22·24%)	745 (27·7%)	0·0061	252 (28·03%)	246 (27·36%)	0·7519	135 (22·24%)	108 (17·79%)	0·0528
Lipid-lowering agents	183 (20·36%)	497 (18·48%)	0·213	136 (22·41%)	497 (18·48%)	0·0264	183 (20·36%)	173 (19·24%)	0·554	136 (22·41%)	128 (21·09%)	0·5778
Anticoagulant	357 (39·71%)	382 (14·2%)	<0·0001	128 (21·09%)	382 (14·2%)	<0·0001	357 (39·71%)	341 (37·93%)	0·4388	128 (21·09%)	120 (19·77%)	0·569
Antiplatelet	401 (44·61%)	1,476 (54·87%)	<0·0001	254 (41·85%)	1,476 (54·87%)	<0·0001	401 (44·61%)	393 (43·72%)	0·704	254 (41·85%)	241 (39·7%)	0·4477
NSAIDs	897 (99·78%)	2,678 (99·55%)	0·3517	606 (99·84%)	2,678 (99·55%)	0·3177	897 (99·78%)	897 (99·78%)	1	606 (99·84%)	606 (99·84%)	1
Medical history												
Liver disease	114 (12·68%)	297 (11·04%)	0·1813	66 (10·87%)	297 (11·04%)	0·9051	114 (12·68%)	98 (10·9%)	0·242	66 (10·87%)	71 (11·7%)	0·6502
Peptic ulcer disease	258 (28·7%)	824 (30·63%)	0·2741	146 (24·05%)	824 (30·63%)	0·0013	258 (28·7%)	245 (27·25%)	0·4946	146 (24·05%)	133 (21·91%)	0·3752
Hypertension	689 (76·64%)	2,188 (81·34%)	0·0022	465 (76·61%)	2,188 (81·34%)	0·0079	689 (76·64%)	676 (75·19%)	0·4734	465 (76·61%)	453 (74·63%)	0·4225
Dyslipidemia	284 (31·59%)	791 (29·41%)	0·2155	206 (33·94%)	791 (29·41%)	0·0281	284 (31·59%)	279 (31·03%)	0·7993	206 (33·94%)	199 (32·78%)	0·67
IHD	371 (41·27%)	1,227 (45·61%)	0·0232	237 (39·04%)	1,227 (45·61%)	0·0033	371 (41·27%)	369 (41·05%)	0·9236	237 (39·04%)	227 (37·4%)	0·5548
ICH	21 (2·34%)	67 (2·49%)	0·795	13 (2·14%)	67 (2·49%)	0·6137	21 (2·34%)	28 (3·11%)	0·3106	13 (2·14%)	15 (2·47%)	0·7022
TIA	46 (5·12%)	125 (4·65%)	0·5669	20 (3·29%)	125 (4·65%)	0·1423	46 (5·12%)	44 (4·89%)	0·8288	20 (3·29%)	14 (2·31%)	0·2966
Alcohol intoxication	8 (0·89%)	22 (0·82%)	0·8373	6 (0·99%)	22 (0·82%)	0·679	8 (0·89%)	4 (0·44%)	0·2466	6 (0·99%)	8 (1·32%)	0·5908
GI bleeding	85 (9·45%)	343 (12·75%)	0·0083	58 (9·56%)	343 (12·75%)	0·0296	85 (9·45%)	67 (7·45%)	0·127	58 (9·56%)	54 (8·9%)	0·6916
Hematuria	34 (3·78%)	94 (3·49%)	0·6873	25 (4·12%)	94 (3·49%)	0·4564	34 (3·78%)	24 (2·67%)	0·182	25 (4·12%)	25 (4·12%)	1
VTE	26 (2·89%)	56 (2·08%)	0·1592	12 (1·98%)	56 (2·08%)	0·8696	26 (2·89%)	18 (2%)	0·2221	12 (1·98%)	9 (1·48%)	0·509
Systemic embolism	26 (2·89%)	94 (3·49%)	0·3845	14 (2·31%)	94 (3·49%)	0·1375	26 (2·89%)	24 (2·67%)	0·7742	14 (2·31%)	8 (1·32%)	0·1967
CHF	464 (51·61%)	1,313 (48·81%)	0·1457	264 (43·49%)	1,313 (48·81%)	0·0178	464 (51·61%)	448 (49·83%)	0·4504	264 (43·49%)	252 (41·52%)	0·486
Myocardial infarction	65 (7·23%)	212 (7·88%)	0·5267	31 (5·11%)	212 (7·88%)	0·0181	65 (7·23%)	72 (8·01%)	0·5338	31 (5·11%)	21 (3·46%)	0·1564
PVD	76 (8·45%)	279 (10·37%)	0·0954	40 (6·59%)	279 (10·37%)	0·0044	76 (8·45%)	66 (7·34%)	0·3819	40 (6·59%)	39 (6·43%)	0·9074
CVA	526 (58·51%)	1,581 (58·77%)	0·8894	406 (66·89%)	1581 (58·77%)	0·0002	526 (58·51%)	517 (57·51%)	0·6672	406 (66·89%)	391 (64·42%)	0·3646
Diabetes mellitus	307 (34·15%)	973 (36·17%)	0·2732	185 (30·48%)	973 (36·17%)	0·008	307 (34·15%)	305 (33·93%)	0·9207	185 (30·48%)	162 (26·69%)	0·144

(*Continued*)

 © 2021. The Author(s).

Supplementary Table S9 (*Continued*)

	Unmatched						Matched					
	Early treatment	No treatment	*p*-Value	Delayed treatment	No treatment	*p*-Value	Early treatment	No treatment	*p*-Value	Delayed treatment	No treatment	*p*-Value
CKD	234 (26·03%)	804 (29·89%)	0·0271	143 (23·56%)	804 (29·89%)	0·0018	234 (26·03%)	234 (26·03%)	1	143 (23·56%)	140 (23·06%)	0·8386
Secondary prevention												
Anticoagulant												
NOAC	176 (19·58%)	0		338 (55·68%)	0		176 (19·58%)	0		338 (55·68%)	0	
Warfarin	622 (69·19%)	0		254 (41·85%)	0		622 (69·19%)	0		254 (41·85%)	0	
Both	101 (11·23%)	0		15 (2·47%)	0		101 (11·23%)	0		15 (2·47%)	0	
Antiplatelet (AIS to index date)	Reference to Supplementary Table 7, severe stroke, 0–12th day for early treatment and 13th–29th day for delayed treatment						668 (74·3%)	662 (73·64%)	0·7471	543 (89·46%)	550 (90·61%)	0·5024

Abbreviations: AIS, acute ischemic stroke; CHF, congestive heart failure; CKD, chronic kidney disease; CVA, cerebrovascular accident; GI, gastrointestinal; ICH, intracranial hemorrhage; IHD, ischemic heart disease; NOAC, non-vitamin K antagonist oral anticoagulant; NSAIDs, nonsteroidal anti-inflammatory drugs; PVD, peripheral vascular disease; TIA, transient ischemic attack; VTE, venous thromboembolism.
Note: Data are mean $\pm$ standard deviation (SD) or *n* (%).

 © 2021. The Author(s).

Supplementary Table S10 Baseline characteristics of mild stroke patients in terms of safety outcomes before and after 30 day-by-day matching

	Unmatched						Matched					
	Early treatment	No treatment	*p*-Value	Delayed treatment	No treatment	*p*-Value	Early treatment	No treatment	*p*-Value	Delayed treatment	No treatment	*p*-Value
Total	1,383 (100%)	1,984 (100%)		1,358 (100%)	1,984 (100%)		1,383 (100%)	1,383 (100%)		1,358 (100%)	1,358 (100%)	
Gender			0·3513			0·3374			0·8485			0·969
Female	624 (45·12%)	863 (43·5%)		568 (41·83%)	863 (43·5%)		624 (45·12%)	629 (45·48%)		568 (41·83%)	569 (41·9%)	
Male	759 (54·88%)	1,121 (56·5%)		790 (58·17%)	1,121 (56·5%)		759 (54·88%)	754 (54·52%)		790 (58·17%)	789 (58·1%)	
Age			<0·0001			<0·0001			0·4701			0·7179
≥80	460 (33·26%)	918 (46·27%)		454 (33·43%)	918 (46·27%)		460 (33·26%)	430 (31·09%)		454 (33·43%)	458 (33·73%)	
70–80	492 (35·57%)	637 (32·11%)		476 (35·05%)	637 (32·11%)		492 (35·57%)	511 (36·95%)		476 (35·05%)	457 (33·65%)	
<70	431 (31·16%)	429 (21·62%)		428 (31·52%)	429 (21·62%)		431 (31·16%)	442 (31·96%)		428 (31·52%)	443 (32·62%)	
Medication history												
Antihypertensive	1,224 (88·5%)	1,732 (87·3%)	0·2934	1,148 (84·54%)	1,732 (87·3%)	0·0231	1,224 (88·5%)	1,238 (89·52%)	0·3947	1,148 (84·54%)	1,118 (82·33%)	0·1216
Antidiabetic	381 (27·55%)	605 (30·49%)	0·0647	344 (25·33%)	605 (30·49%)	0·0012	381 (27·55%)	374 (27·04%)	0·7651	344 (25·33%)	353 (25·99%)	0·6926
Lipid-lowering agents	326 (23·57%)	426 (21·47%)	0·15	318 (23·42%)	426 (21·47%)	0·1843	326 (23·57%)	344 (24·87%)	0·4244	318 (23·42%)	312 (22·97%)	0·785
Anticoagulant	661 (47·79%)	210 (10·58%)	<0·0001	320 (23·56%)	210 (10·58%)	<0·0001	661 (47·79%)	643 (46·49%)	0·493	320 (23·56%)	277 (20·4%)	0·0463
Antiplatelet	641 (46·35%)	1,198 (60·38%)	<0·0001	722 (53·17%)	1,198 (60·38%)	<0·0001	641 (46·35%)	681 (49·24%)	0·1279	722 (53·17%)	722 (53·17%)	1
NSAIDs	1,383 (100%)	1,981 (99·85%)	1	1,358 (100%)	1,981 (99·85%)	1	1,383 (100%)	1,383 (100%)	0·5388	1,358 (100%)	1,358 (100%)	0·5388
Medical history												
Liver disease	189 (13·67%)	302 (15·22%)	0·2082	221 (16·27%)	302 (15·22%)	0·4109	189 (13·67%)	192 (13·88%)	0·8685	221 (16·27%)	208 (15·32%)	0·494
Peptic ulcer disease	374 (27·04%)	695 (35·03%)	<0·0001	386 (28·42%)	695 (35·03%)	<0·0001	374 (27·04%)	359 (25·96%)	0·5181	386 (28·42%)	391 (28·79%)	0·8319
Hypertension	1,084 (78·38%)	1,666 (83·97%)	<0·0001	1,065 (78·42%)	1,666 (83·97%)	<0·0001	1,084 (78·38%)	1,103 (79·75%)	0·3745	1,065 (78·42%)	1,082 (79·68%)	0·4228
Dyslipidemia	484 (35%)	688 (34·68%)	0·8484	491 (36·16%)	688 (34·68%)	0·3796	484 (35%)	478 (34·56%)	0·8107	491 (36·16%)	487 (35·86%)	0·873
IHD	662 (47·87%)	1,005 (50·66%)	0·1114	643 (47·35%)	1,005 (50·66%)	0·0604	662 (47·87%)	670 (48·45%)	0·7608	643 (47·35%)	658 (48·45%)	0·5645
ICH	28 (2·02%)	64 (3·23%)	0·0354	21 (1·55%)	64 (3·23%)	0·0025	28 (2·02%)	34 (2·46%)	0·4409	21 (1·55%)	17 (1·25%)	0·5135
TIA	101 (7·3%)	145 (7·31%)	0·9952	97 (7·14%)	145 (7·31%)	0·856	101 (7·3%)	86 (6·22%)	0·256	97 (7·14%)	89 (6·55%)	0·5433
Alcohol intoxication	13 (0·94%)	24 (1·21%)	0·4602	12 (0·88%)	24 (1·21%)	0·3699	13 (0·94%)	13 (0·94%)	1	12 (0·88%)	15 (1·1%)	0·5618
GI bleeding	129 (9·33%)	275 (13·86%)	<0·0001	127 (9·35%)	275 (13·86%)	<0·0001	129 (9·33%)	122 (8·82%)	0·6431	127 (9·35%)	129 (9·5%)	0·8955
Hematuria	70 (5·06%)	92 (4·64%)	0·5713	67 (4·93%)	92 (4·64%)	0·6924	70 (5·06%)	71 (5·13%)	0·9311	67 (4·93%)	59 (4·34%)	0·4655
VTE	30 (2·17%)	30 (1·51%)	0·1562	25 (1·84%)	30 (1·51%)	0·463	30 (2·17%)	25 (1·81%)	0·4959	25 (1·84%)	19 (1·4%)	0·3618
Systemic embolism	35 (2·53%)	70 (3·53%)	0·1014	48 (3·53%)	70 (3·53%)	0·9922	35 (2·53%)	35 (2·53%)	1	48 (3·53%)	38 (2·8%)	0·2732
CHF	640 (46·28%)	884 (44·56%)	0·324	531 (39·1%)	884 (44·56%)	0·0017	640 (46·28%)	612 (44·25%)	0·2848	531 (39·1%)	512 (37·7%)	0·4535
Myocardial infarction	109 (7·88%)	201 (10·13%)	0·0263	96 (7·07%)	201 (10·13%)	0·0022	109 (7·88%)	103 (7·45%)	0·668	96 (7·07%)	97 (7·14%)	0·9405
PVD	120 (8·68%)	208 (10·48%)	0·0819	110 (8·1%)	208 (10·48%)	0·0211	120 (8·68%)	123 (8·89%)	0·8403	110 (8·1%)	98 (7·22%)	0·3866
CVA	643 (46·49%)	926 (46·67%)	0·9178	650 (47·86%)	926 (46·67%)	0·4981	643 (46·49%)	614 (44·4%)	0·2681	650 (47·86%)	625 (46·02%)	0·3364
Diabetes mellitus	478 (34·56%)	790 (39·82%)	0·002	454 (33·43%)	790 (39·82%)	0·0002	478 (34·56%)	455 (32·9%)	0·355	454 (33·43%)	432 (31·81%)	0·3679

(*Continued*)

 © 2021. The Author(s).

Supplementary Table S10 (*Continued*)

		Unmatched					Matched					
	Early treatment	No treatment	*p*-Value	Delayed treatment	No treatment	*p*-Value	Early treatment	No treatment	*p*-Value	Delayed treatment	No treatment	*p*-Value
CKD	377 (27·26%)	680 (34·27%)	<0·0001	340 (25·04%)	680 (34·27%)	<·0001	377 (27·26%)	383 (27·69%)	0·7983	340 (25·04%)	321 (23·64%)	0·3956
Secondary prevention												
Anticoagulant												
NOAC	373 (26·97%)	0		762 (56·11%)	0		373 (26·97%)	0		762 (56·11%)	0	
Warfarin	845 (61·1%)	0		530 (39·03%)	0		845 (61·1%)	0		530 (39·03%)	0	
Both	165 (11·93%)	0		66 (4·86%)	0		165 (11·93%)	0		66 (4·86%)	0	
Antiplatelet (AIS to index date)	Reference to Supplementary Table 11, mild stroke, 0–3rd day for early treatment and 4th–29th day for delayed treatment						884 (63·92%)	868 (62·76%)	0·5278	1,153 (84·9%)	1,163 (85·64%)	0·5882

Abbreviations: AIS, acute ischemic stroke; CHF, congestive heart failure; CKD, chronic kidney disease; CVA, cerebrovascular accident; GI, gastrointestinal; ICH, intracranial hemorrhage; IHD, ischemic heart disease; NOAC, non-vitamin K antagonist oral anticoagulant; NSAIDs, nonsteroidal anti-inflammatory drugs; PVD, peripheral vascular disease; TIA, transient ischemic attack; VTE, venous thromboembolism.
Note: Data are mean ± standard deviation (SD) or *n* (%).

 © 2021. The Author(s).

Supplementary Table S11 Use of antiplatelet treatment for stroke of varying severity in terms of the safety outcome before day-by-day matching

	Mild				Moderate				Severe			
	Use of anticoagulants		No use of anticoagulants		Use of anticoagulants		No use of anticoagulants		Use of anticoagulants		No use of anticoagulants	
Day	*N*	Use of antiplatelet	*N*	Use of antiplatelet	*N*	Use of antiplatelet	*N*	Use of antiplatelet	*N*	Use of antiplatelet	*N*	Use of antiplatelet
0	577	296 (51·3%)	4,148	3,396 (81·87%)	208	119 (57·21%)	2,547	2,102 (82·53%)	205	130 (63·41%)	4,024	3,446 (85·64%)
1	420	275 (65·48%)	3,723	3,119 (83·78%)	189	115 (60·85%)	2,335	1,975 (84·58%)	166	120 (72·29%)	3,817	3,302 (86·51%)
2	211	168 (79·62%)	3,508	2,954 (84·21%)	107	81 (75·7%)	2,212	1,880 (84·99%)	93	63 (67·74%)	3,660	3,195 (87·3%)
3	175	144 (82·29%)	3,330	2,810 (84·38%)	82	54 (65·85%)	2,111	1,814 (85·93%)	61	46 (75·41%)	3,510	3,081 (87·78%)
4	142	121 (85·21%)	3,187	2,690 (84·41%)	71	59 (83·1%)	2,017	1,737 (86·12%)	66	45 (68·18%)	3,383	2,992 (88·44%)
5	100	77 (77%)	3,083	2,610 (84·66%)	46	40 (86·96%)	1,962	1,691 (86·19%)	50	33 (66%)	3,267	2,904 (88·89%)
6	97	73 (75·26%)	2,984	2,539 (85·09%)	61	47 (77·05%)	1,893	1,637 (86·48%)	50	43 (86%)	3,182	2,833 (89·03%)
7	97	79 (81·44%)	2,885	2,458 (85·2%)	58	49 (84·48%)	1,823	1,578 (86·56%)	36	31 (86·11%)	3,116	2,777 (89·12%)
8	66	52 (78·79%)	2,818	2,408 (85·45%)	45	43 (95·56%)	1,768	1,528 (86·43%)	50	44 (88%)	3,032	2,708 (89·31%)
9	63	57 (90·48%)	2,749	2,354 (85·63%)	37	35 (94·59%)	1,719	1,486 (86·45%)	37	33 (89·19%)	2,963	2,650 (89·44%)
10	70	63 (90%)	2,674	2,292 (85·71%)	41	38 (92·68%)	1,673	1,445 (86·37%)	52	48 (92·31%)	2,883	2,580 (89·49%)
11	84	70 (83·33%)	2,583	2,221 (85·99%)	40	34 (85%)	1,627	1,409 (86·6%)	46	41 (89·13%)	2,811	2,518 (89·58%)
12	69	60 (86·96%)	2,501	2,154 (86·13%)	52	47 (90·38%)	1,566	1,355 (86·53%)	40	34 (85%)	2,748	2,466 (89·74%)
13	78	69 (88·46%)	2,412	2,083 (86·36%)	51	46 (90·2%)	1,507	1,305 (86·6%)	65	54 (83·08%)	2,671	2,401 (89·89%)
14	107	92 (85·98%)	2,298	1,990 (86·6%)	71	65 (91·55%)	1,430	1,234 (86·29%)	105	96 (91·43%)	2,541	2,286 (89·96%)
15	63	61 (96·83%)	2,227	1,927 (86·53%)	41	39 (95·12%)	1,379	1,186 (86%)	66	57 (86·36%)	2,450	2,205 (90%)
16	50	43 (86%)	2,173	1,884 (86·7%)	42	40 (95·24%)	1,329	1,140 (85·78%)	55	47 (85·45%)	2,380	2,149 (90·29%)
17	37	34 (91·89%)	2,128	1,845 (86·7%)	26	21 (80·77%)	1,294	1,117 (86·32%)	53	45 (84·91%)	2,308	2,088 (90·47%)
18	26	22 (84·62%)	2,094	1,823 (87·06%)	28	27 (96·43%)	1,258	1,087 (86·41%)	40	37 (92·5%)	2,245	2,028 (90·33%)
19	27	25 (92·59%)	2,062	1,794 (87%)	28	24 (85·71%)	1,220	1,058 (86·72%)	45	40 (88·89%)	2,185	1,976 (90·43%)
20	24	20 (83·33%)	2,034	1,775 (87·27%)	16	13 (81·25%)	1,193	1,035 (86·76%)	35	33 (94·29%)	2,139	1,934 (90·42%)
21	31	23 (74·19%)	1,996	1,748 (87·58%)	12	7 (58·33%)	1,175	1,023 (87·06%)	23	17 (73·91%)	2,105	1,910 (90·74%)
22	21	20 (95·24%)	1,972	1,731 (87·78%)	10	10 (100%)	1,160	1009 (86·98%)	21	18 (85·71%)	2,074	1,886 (90·94%)
23	21	17 (80·95%)	1,947	1,714 (88·03%)	16	14 (87·5%)	1,141	994 (87·12%)	25	25 (100%)	2,038	1,852 (90·87%)
24	16	14 (87·5%)	1,925	1,695 (88·05%)	5	3 (60%)	1,124	984 (87·54%)	20	18 (90%)	2,003	1,820 (90·86%)
25	13	11 (84·62%)	1,911	1,683 (88·07%)	10	8 (80%)	1,107	969 (87·53%)	22	21 (95·45%)	1,968	1,789 (90·9%)
26	19	17 (89·47%)	1,888	1,667 (88·29%)	9	8 (88·89%)	1,098	961 (87·52%)	14	13 (92·86%)	1,946	1,769 (90·9%)
27	15	10 (66·67%)	1,869	1,656 (88·6%)	4	3 (75%)	1,087	953 (87·67%)	14	12 (85·71%)	1,912	1,739 (90·95%)
28	12	12 (100%)	1,851	1,643 (88·76%)	10	10 (100%)	1,074	943 (87·8%)	19	17 (89·47%)	1,882	1,717 (91·23%)
29	10	6 (60%)	1,839	1,638 (89·07%)	9	8 (88·89%)	1,056	930 (88·07%)	21	19 (90·48%)	1,840	1,678 (91·2%)

 © 2021. The Author(s).

Supplementary Table S12 Baseline characteristics of moderate stroke patients about safety outcome before and after 30 day-by-day matching

	Unmatched						Matched					
	Early treatment	No treatment	*p*-Value	Delayed treatment	No treatment	*p*-Value	Early treatment	No treatment	*p*-Value	Delayed treatment	No treatment	*p*-Value
Total	764 (100%)	1,330 (100%)		661 (100%)	1,330 (100%)		763 (100%)	763 (100%)		658 (100%)	658 (100%)	
Gender			0·2922			0·0495			0·7979			0·6995
Female	385 (50·39%)	702 (52·78%)		318 (48·11%)	702 (52·78%)		385 (50·46%)	390 (51·11%)		318 (48·33%)	325 (49·39%)	
Male	379 (49·61%)	628 (47·22%)		343 (51·89%)	628 (47·22%)		378 (49·54%)	373 (48·89%)		340 (51·67%)	333 (50·61%)	
Age			<0·0001			<0·0001			0·185			0·6219
≥80	296 (38·74%)	809 (60·83%)		259 (39·18%)	809 (60·83%)		296 (38·79%)	298 (39·06%)		258 (39·21%)	246 (37·39%)	
70–80	248 (32·46%)	339 (25·49%)		197 (29·8%)	339 (25·49%)		248 (32·5%)	219 (28·7%)		197 (29·94%)	213 (32·37%)	
<70	220 (28·8%)	182 (13·68%)		205 (31·01%)	182 (13·68%)		219 (28·7%)	246 (32·24%)		203 (30·85%)	199 (30·24%)	
Medication history												
Antihypertensive	673 (88·09%)	1,124 (84·51%)	0·0239	563 (85·17%)	1124 (84·51%)	0·6986	672 (88·07%)	670 (87·81%)	0·8751	560 (85·11%)	563 (85·56%)	0·8152
Antidiabetic	198 (25·92%)	357 (26·84%)	0·644	157 (23·75%)	357 (26·84%)	0·1379	198 (25·95%)	178 (23·33%)	0·2348	157 (23·86%)	154 (23·4%)	0·8457
Lipid-lowering agents	157 (20·55%)	236 (17·74%)	0·1135	125 (18·91%)	236 (17·74%)	0·5247	156 (20·45%)	156 (20·45%)	1	123 (18·69%)	122 (18·54%)	0·9435
Anticoagulant	312 (40·84%)	156 (11·73%)	<0·0001	142 (21·48%)	156 (11·73%)	<0·0001	311 (40·76%)	302 (39·58%)	0·6384	140 (21·28%)	132 (20·06%)	0·586
Antiplatelet	326 (42·67%)	763 (57·37%)	<0·0001	317 (47·96%)	763 (57·37%)	<0·0001	326 (42·73%)	335 (43·91%)	0·642	316 (48·02%)	292 (44·38%)	0·1845
NSAIDs	764 (100%)	1,327 (99·77%)	1	660 (99·85%)	1327 (99·77%)	0·7274	763 (100%)	763 (100%)	0·5388	657 (99·85%)	657 (99·85%)	1
Medical history												
Liver disease	98 (12·83%)	177 (13·31%)	0·7537	89 (13·46%)	177 (13·31%)	0·9232	98 (12·84%)	92 (12·06%)	0·6418	88 (13·37%)	90 (13·68%)	0·8719
Peptic ulcer disease	206 (26·96%)	439 (33·01%)	0·0039	196 (29·65%)	439 (33·01%)	0·1303	206 (27%)	196 (25·69%)	0·5611	194 (29·48%)	180 (27·36%)	0·3922
Hypertension	579 (75·79%)	1,091 (82·03%)	0·0006	502 (75·95%)	1091 (82·03%)	0·0014	579 (75·88%)	586 (76·8%)	0·6733	499 (75·84%)	487 (74·01%)	0·4454
Dyslipidemia	261 (34·16%)	374 (28·12%)	0·0038	222 (33·59%)	374 (28·12%)	0·0122	261 (34·21%)	267 (34·99%)	0·7468	221 (33·59%)	221 (33·59%)	1
IHD	328 (42·93%)	616 (46·32%)	0·1341	309 (46·75%)	616 (46·32%)	0·8557	328 (42·99%)	337 (44·17%)	0·6422	307 (46·66%)	311 (47·26%)	0·8251
ICH	11 (1·44%)	53 (3·98%)	0·0011	15 (2·27%)	53 (3·98%)	0·0471	11 (1·44%)	15 (1·97%)	0·4288	15 (2·28%)	15 (2·28%)	1
TIA	40 (5·24%)	75 (5·64%)	0·6964	36 (5·45%)	75 (5·64%)	0·8598	40 (5·24%)	44 (5·77%)	0·6535	35 (5·32%)	32 (4·86%)	0·7068
Alcohol intoxication	4 (0·52%)	14 (1·05%)	0·2068	9 (1·36%)	14 (1·05%)	0·5435	4 (0·52%)	7 (0·92%)	0·364	8 (1·22%)	6 (0·91%)	0·591
GI bleeding	83 (10·86%)	197 (14·81%)	0·0106	73 (11·04%)	197 (14·81%)	0·0207	83 (10·88%)	71 (9·31%)	0·3078	73 (11·09%)	64 (9·73%)	0·4166
Hematuria	36 (4·71%)	75 (5·64%)	0·362	30 (4·54%)	75 (5·64%)	0·3008	36 (4·72%)	40 (5·24%)	0·6379	30 (4·56%)	27 (4·1%)	0·6846
VTE	27 (3·53%)	29 (2·18%)	0·0646	10 (1·51%)	29 (2·18%)	0·3114	26 (3·41%)	29 (3·8%)	0·6803	10 (1·52%)	11 (1·67%)	0·8259
Systemic embolism	16 (2·09%)	53 (3·98%)	0·0196	17 (2·57%)	53 (3·98%)	0·1069	16 (2·1%)	16 (2·1%)	1	16 (2·43%)	13 (1·98%)	0·5732
CHF	378 (49·48%)	649 (48·8%)	0·7646	293 (44·33%)	649 (48·8%)	0·0599	377 (49·41%)	390 (51·11%)	0·5057	291 (44·22%)	295 (44·83%)	0·8244
Myocardial infarction	43 (5·63%)	129 (9·7%)	0·0011	51 (7·72%)	129 (9·7%)	0·1461	43 (5·64%)	35 (4·59%)	0·3524	51 (7·75%)	46 (6·99%)	0·5979
PVD	65 (8·51%)	134 (10·08%)	0·2391	47 (7·11%)	134 (10·08%)	0·0302	65 (8·52%)	61 (7·99%)	0·7099	46 (6·99%)	37 (5·62%)	0·3075
CVA	408 (53·4%)	703 (52·86%)	0·8096	342 (51·74%)	703 (52·86%)	0·6382	407 (53·34%)	417 (54·65%)	0·6075	339 (51·52%)	327 (49·7%)	0·5082
Diabetes mellitus	257 (33·64%)	484 (36·39%)	0·2048	215 (32·53%)	484 (36·39%)	0·0889	257 (33·68%)	242 (31·72%)	0·4131	215 (32·67%)	221 (33·59%)	0·7253

 © 2021. The Author(s).

Supplementary Table S12 (*Continued*)

	Unmatched						Matched					
	Early treatment	No treatment	*p*-Value	Delayed treatment	No treatment	*p*-Value	Early treatment	No treatment	*p*-Value	Delayed treatment	No treatment	*p*-Value
CKD	210 (27·49%)	442 (33·23%)	0·0063	182 (27·53%)	442 (33·23%)	0·0098	210 (27·52%)	209 (27·39%)	0·9543	182 (27·66%)	192 (29·18%)	0·5411
Secondary prevention												
Anticoagulant												
NOAC	183 (23·95%)	0		367 (55·52%)	0		183 (23·98%)	0		365 (55·47%)	0	
Warfarin	469 (61·39%)	0		267 (40·39%)	0		468 (61·34%)	0		266 (40·43%)	0	
Both	112 (14·66%)	0		27 (4·08%)	0		112 (14·68%)	0		27 (4·1%)	0	
Antiplatelet (AIS to index date)	Reference to Supplementary Table 11, moderate stroke, 0–6th day for early treatment and 7th–29th day for delayed treatment						515 (67·5%)	510 (66·84%)	0·7852	590 (89·67%)	593 (90·12%)	0·7838

Abbreviations: AIS, acute ischemic stroke; CHF, congestive heart failure; CKD, chronic kidney disease; CVA, cerebrovascular accident; GI, gastrointestinal; ICH, intracranial hemorrhage; IHD, ischemic heart disease; NOAC, non-vitamin K antagonist oral anticoagulant; NSAIDs, nonsteroidal anti-inflammatory drugs; PVD, peripheral vascular disease; TIA, transient ischemic attack; VTE, venous thromboembolism.
Note: Data are mean $\pm$ standard deviation (SD) or *n* (%).

 © 2021. The Author(s).

Supplementary Table S13 Baseline characteristics of severe stroke patients in terms of the safety outcome before and after 30 day-by-day matching

	Unmatched						Matched					
	Early treatment	No treatment	*p*-Value	Delayed treatment	No treatment	*p*-Value	Early treatment	No treatment	*p*-Value	Delayed treatment	No treatment	*p*-Value
Total	952 (100%)	2,634 (100%)		643 (100%)	2,634 (100%)		950 (100%)	950 (100%)		642 (100%)	642 (100%)	
Gender			0·9333			0·9311			0·7441			0·9549
Female	557 (58·51%)	1,537 (58·35%)		374 (58·16%)	1,537 (58·35%)		556 (58·53%)	563 (59·26%)		373 (58·1%)	372 (57·94%)	
Male	395 (41·49%)	1,097 (41·65%)		269 (41·84%)	1,097 (41·65%)		394 (41·47%)	387 (40·74%)		269 (41·9%)	270 (42·06%)	
Age			<0·0001			<0·0001			0·8183			0·5933
≥80	386 (40·55%)	1,382 (52·47%)		263 (40·9%)	1,382 (52·47%)		386 (40·63%)	393 (41·37%)		263 (40·97%)	263 (40·97%)	
70–80	329 (34·56%)	808 (30·68%)		216 (33·59%)	808 (30·68%)		328 (34·53%)	315 (33·16%)		215 (33·49%)	201 (31·31%)	
<70	237 (24·89%)	444 (16·86%)		164 (25·51%)	444 (16·86%)		236 (24·84%)	242 (25·47%)		164 (25·55%)	178 (27·73%)	
Medication history												
Antihypertensive	840 (88·24%)	2,288 (86·86%)	0·2773	553 (86%)	2,288 (86·86%)	0·5644	838 (88·21%)	837 (88·11%)	0·9434	552 (85·98%)	552 (85·98%)	1
Antidiabetic	265 (27·84%)	744 (28·25%)	0·8095	144 (22·4%)	744 (28·25%)	0·0028	264 (27·79%)	251 (26·42%)	0·5023	144 (22·43%)	130 (20·25%)	0·3403
Lipid-lowering agents	200 (21·01%)	508 (19·29%)	0·2526	146 (22·71%)	508 (19·29%)	0·0518	198 (20·84%)	175 (18·42%)	0·184	145 (22·59%)	139 (21·65%)	0·6866
Anticoagulant	372 (39·08%)	381 (14·46%)	<0·0001	135 (21%)	381 (14·46%)	<0·0001	370 (38·95%)	365 (38·42%)	0·8138	134 (20·87%)	137 (21·34%)	0·8374
Antiplatelet	430 (45·17%)	1,470 (55·81%)	<0·0001	283 (44·01%)	1,470 (55·81%)	<0·0001	429 (45·16%)	444 (46·74%)	0·4899	282 (43·93%)	273 (42·52%)	0·6121
NSAID	950 (99·79%)	2,621 (99·51%)	0·2455	641 (99·69%)	2,621 (99·51%)	0·5388	948 (99·79%)	948 (99·79%)	1	640 (99·69%)	640 (99·69%)	1
Medical history												
Liver disease	116 (12·18%)	295 (11·2%)	0·4135	71 (11·04%)	295 (11·2%)	0·9094	115 (12·11%)	104 (10·95%)	0·4294	70 (10·9%)	65 (10·12%)	0·6492
Peptic ulcer disease	263 (27·63%)	817 (31·02%)	0·0506	153 (23·79%)	817 (31·02%)	0·0003	263 (27·68%)	262 (27·58%)	0·9591	152 (23·68%)	135 (21·03%)	0·2548
Hypertension	737 (77·42%)	2,156 (81·85%)	0·003	497 (77·29%)	2,156 (81·85%)	0·0083	735 (77·37%)	738 (77·68%)	0·869	496 (77·26%)	512 (79·75%)	0·277
Dyslipidemia	311 (32·67%)	789 (29·95%)	0·1197	214 (33·28%)	789 (29·95%)	0·1008	309 (32·53%)	274 (28·84%)	0·0817	213 (33·18%)	207 (32·24%)	0·7212
IHD	396 (41·6%)	1,259 (47·8%)	0·001	267 (41·52%)	1,259 (47·8%)	0·0042	395 (41·58%)	400 (42·11%)	0·8161	266 (41·43%)	255 (39·72%)	0·5319
ICH	23 (2·42%)	69 (2·62%)	0·7334	13 (2·02%)	69 (2·62%)	0·3842	23 (2·42%)	24 (2·53%)	0·8826	13 (2·02%)	11 (1·71%)	0·6803
TIA	47 (4·94%)	125 (4·75%)	0·8128	22 (3·42%)	125 (4·75%)	0·1459	46 (4·84%)	51 (5·37%)	0·6023	22 (3·43%)	17 (2·65%)	0·4162
Alcohol intoxication	7 (0·74%)	22 (0·84%)	0·7679	6 (0·93%)	22 (0·84%)	0·8089	6 (0·63%)	3 (0·32%)	0·3162	6 (0·93%)	8 (1·25%)	0·5909
GI bleeding	80 (8·4%)	305 (11·58%)	0·0067	50 (7·78%)	305 (11·58%)	0·0054	80 (8·42%)	61 (6·42%)	0·0963	49 (7·63%)	50 (7·79%)	0·9167
Hematuria	37 (3·89%)	89 (3·38%)	0·4659	25 (3·89%)	89 (3·38%)	0·5276	37 (3·89%)	35 (3·68%)	0·8101	24 (3·74%)	21 (3·27%)	0·6489
VTE	28 (2·94%)	63 (2·39%)	0·3556	14 (2·18%)	63 (2·39%)	0·7475	28 (2·95%)	20 (2·11%)	0·2422	14 (2·18%)	14 (2·18%)	1
Systemic embolism	38 (3·99%)	111 (4·21%)	0·7681	19 (2·95%)	111 (4·21%)	0·1425	38 (4%)	36 (3·79%)	0·8125	19 (2·96%)	14 (2·18%)	0·3779
CHF	505 (53·05%)	1,331 (50·53%)	0·1834	285 (44·32%)	1,331 (50·53%)	0·0048	503 (52·95%)	487 (51·26%)	0·4625	285 (44·39%)	291 (45·33%)	0·7364
Myocardial infarction	85 (8·93%)	256 (9·72%)	0·4761	43 (6·69%)	256 (9·72%)	0·0167	85 (8·95%)	89 (9·37%)	0·7504	43 (6·7%)	43 (6·7%)	1
PVD	94 (9·87%)	289 (10·97%)	0·3472	45 (7%)	289 (10·97%)	0·0028	93 (9·79%)	89 (9·37%)	0·7552	45 (7·01%)	40 (6·23%)	0·5746
CVA	549 (57·67%)	1,570 (59·61%)	0·2975	415 (64·54%)	1,570 (59·61%)	0·0217	548 (57·68%)	544 (57·26%)	0·8527	414 (64·49%)	421 (65·58%)	0·6821
Diabetes mellitus	326 (34·24%)	978 (37·13%)	0·1126	196 (30·48%)	978 (37·13%)	0·0016	324 (34·11%)	297 (31·26%)	0·1866	196 (30·53%)	182 (28·35%)	0·3913

 © 2021. The Author(s).

Supplementary Table S13 (*Continued*)

	Unmatched						Matched					
	Early treatment	No treatment	*p*-Value	Delayed treatment	No treatment	*p*-Value	Early treatment	No treatment	*p*-Value	Delayed treatment	No treatment	*p*-Value
CKD	240 (25·21%)	808 (30·68%)	0·0015	151 (23·48%)	808 (30·68%)	0·0003	239 (25·16%)	223 (23·47%)	0·3922	150 (23·36%)	143 (22·27%)	0·6416
Secondary prevention												
Anticoagulant												
NOAC	179 (18·8%)	0		347 (53·97%)	0		179 (18·84%)	0		347 (54·05%)	0	
Warfarin	662 (69·54%)	0		271 (42·15%)	0		660 (69·47%)	0		270 (42·06%)	0	
Both	111 (11·66%)	0		25 (3·89%)	0		111 (11·68%)	0		25 (3·89%)	0	
Antiplatelet (AIS to index date)	Reference to Supplementary Table 11, severe stroke, 0–12th day for early treatment and 13th–29th day for delayed treatment						711 (74·84%)	721 (75·89%)	0·5944	573 (89·25%)	580 (90·34%)	0·5187

Abbreviations: AIS, acute ischemic stroke; CHF, congestive heart failure; CKD, chronic kidney disease; CVA, cerebrovascular accident; GI, gastrointestinal; ICH, intracranial hemorrhage; IHD, ischemic heart disease; NOAC, non-vitamin K antagonist oral anticoagulant; NSAIDs, nonsteroidal anti-inflammatory drugs; PVD, peripheral vascular disease; TIA, transient ischemic attack; VTE, venous thromboembolism.
Note: Data are mean ± standard deviation (SD) or *n* (%).

 © 2021. The Author(s).

Supplementary Table S14 Incidence rate, rate ratio (RR), rate difference (RD), and number needed to treat (NNT) for composite outcome, effectiveness, and safety, in early use and delayed use of OACs in patients with AF hospitalized for stroke

	AF patient with mild stroke				AF patient with moderate stroke				AF patient with severe stroke			
	OACs use in PS-matched cohorts											
	Early	No	Delayed	No	Early	No	Delayed	No	Early	No	Delayed	No
Composite outcome												
Case number	724	321	597	426	425	207	322	217	549	362	354	255
Person-years (p-yr)	1,714·48	437·23	1,536·26	964·96	740·69	175·7	671·33	350·23	584·77	262·67	477·34	312·66
Severity-specific incidence rate, per 1,000 p-yr	444·5				604·25				928·28			
Incidence rate, per 1,000 p-yr	422·29	734·16	388·61	441·47	573·79	1,178·17	479·64	619·59	938·83	1,378·14	741·61	815·58
Incidence RD, per 1,000 p-yr	311·87	Ref.	52·86	Ref.	604·38	Ref.	139·95	Ref.	439·31	Ref.	73·97	Ref.
Incidence RR (95% CI)	0·58 (0·53, 0·62)	Ref.	0·88 (0·81, 0·95)	Ref.	0·49 (0·44, 0·54)	Ref.	0·77 (0·69, 0·87)	Ref.	0·68 (0·62, 0·75)	Ref.	0·91 (0·81, 1·02)	Ref.
NNT	3·2		18·9		1·7		7·1		2·3		13·5	
Effectiveness outcome												
Case number	570	250	447	352	356	179	269	203	487	330	321	249
Person-years (p-yr)	2,009·03	600·59	1,813·92	1,119·64	908·59	280·56	814·21	448·09	742·88	365·07	597·77	412·7
Stroke severity-specific incidence rate, per 1,000 p-yr	292·07				410·78				654·73			
Incidence rate, per 1,000 p-yr	283·72	416·26	246·43	314·39	391·82	638	330·38	453·03	655·56	903·93	537	603·34
Incidence RD, per 1,000 p-yr	132·54	Ref.	67·96	Ref.	246·18	Ref.	122·65	Ref.	248·37	Ref.	66·34	Ref.
Incidence RR (95% CI)	0·68 (0·63, 0·74)	Ref.	0·78 (0·72, 0·85)	Ref.	0·61 (0·55, 0·68)	Ref.	0·73 (0·65, 0·82)	Ref.	0·73 (0·66, 0·80)	Ref.	0·89 (0·80, 1·00)	Ref.
NNT	7·5		14·7		4·1		8·2		4·0		15·1	
Safety outcome												
Case number	349	119	330	226	190	68	170	96	219	95	141	99
Person-years (p-yr)	2,493·64	753·3	2,245·76	1,356·68	1,152·07	323·82	1,021·38	445·74	1,032·39	450·05	853·55	485
Stroke severity-specific incidence rate, per 1,000 p-yr	149·50				178·05				196·38			
Incidence rate, per 1,000 p-yr	139·96	157·97	146·94	166·58	164·92	209·99	166·44	215·37	212·13	211·09	165·19	204·12
Incidence RD, per 1,000 p-yr	18·01	Ref.	19·64	Ref.	45·07	Ref.	48·93	Ref.	−1·04	Ref.	38·93	Ref.
Incidence RR (95% CI)	0·89 (0·82, 0·95)	Ref.	0·88 (0·82, 0·95)	Ref.	0·79 (0·71, 0·87)	Ref.	0·77 (0·69, 0·86)	Ref.	1·00 (0·92, 1·10)	Ref.	0·81 (0·73, 0·90)	Ref.
NNT	55·5		50·9		22·2		20·4		−961·5		25·7	

Abbreviations: AF, atrial fibrillation; OAC, oral anticoagulant.

 © 2021. The Author(s).

Supplementary Table S15 Descriptive results of the mild stroke in terms of composite outcomes

	Mild stroke			
	Early treatment		Delayed treatment	
Composite outcome	User	Nonuser	User	Nonuser
Sample, *n*	1,307	1,307	1,171	1,171
Observed events, *n* (%)	724 (55·39%)	321 (24·56%)	597 (50·98%)	426 (36·38%)
Ischemic stroke	208 (15·91%)	122 (9·33%)	215 (18·36%)	135 (11·53%)
Gastrointestinal bleeding	150 (11·48%)	57 (4·36%)	125 (10·67%)	95 (8·11%)
Transient ischemic attack	104 (7·96%)	48 (3·67%)	44 (3·76%)	57 (4·87%)
Myocardial infarction	81 (6·2%)	29 (2·22%)	53 (4·53%)	45 (3·84%)
Hematuria	59 (4·51%)	24 (1·84%)	72 (6·15%)	25 (2·13%)
Cardiovascular death	49 (3·75%)	20 (1·53%)	44 (3·76%)	43 (3·67%)
Systemic embolism	28 (2·14%)	13 (0·99%)	16 (1·37%)	16 (1·37%)
Venous thromboembolism	27 (2·07%)	6 (0·46%)	15 (1·28%)	8 (0·68%)
Intracranial hemorrhage	18 (1·38%)	2 (0·15%)	13 (1·11%)	2 (0·17%)
Competing risk events, *n* (%)	38 (2·91%)	40 (3·06%)	47 (4·01%)	57 (4·87%)
Drug censored[a], *n* (%)	249 (19·05%)	180 (13·77%)	222 (18·96%)	266 (22·72%)
Pair censored[b], *n* (%)		713 (54·55%)		295 (25·19%)
Median of follow-up days (IQR)	256 (45–743)	13 (4–61)	308 (60–732)	52 (10–459)
Total follow-up year	1,714·48	437·23	1,536·26	964·96
Crude incidence rate (1,000 person-years)	422·29	734·16	388·61	441·47
Difference in incidence rate	311·87		52·86	
Ratio of incidence rate (95% CI)	0·58 (0·53, 0·62)		0·88 (0·81, 0·95)	

Abbreviations: CI, confidence interval; IQR, interquartile range.
[a]Censored caused by holding or use of drugs.
[b]Use of drug within 30 days but categorized as controlled group owing to paired matching.

 © 2021. The Author(s).

Supplementary Table S16 Descriptive results of the moderate stroke in terms of composite outcomes

	Moderate stroke			
	Early treatment		Delayed treatment	
**Composite outcome**	**User**	**Nonuser**	**User**	**Nonuser**
Sample, *n*	691	691	572	572
Observed events, *n* (%)	425 (61·51%)	207 (29·96%)	322 (56·29%)	217 (37·94%)
Ischemic stroke	153 (22·14%)	98 (14·18%)	133 (23·25%)	81 (14·16%)
Gastrointestinal bleeding	77 (11·14%)	33 (4·78%)	49 (8·57%)	45 (7·87%)
Transient ischemic attack	69 (9·99%)	44 (6·37%)	36 (6·29%)	48 (8·39%)
Myocardial infarction	34 (4·92%)	5 (0·72%)	35 (6·12%)	7 (1·22%)
Hematuria	26 (3·76%)	13 (1·88%)	20 (3·5%)	13 (2·27%)
Cardiovascular death	32 (4·63%)	5 (0·72%)	25 (4·37%)	13 (2·27%)
Systemic embolism	7 (1·01%)	6 (0·87%)	7 (1·22%)	6 (1·05%)
Venous thromboembolism	18 (2·6%)	1 (0·14%)	7 (1·22%)	3 (0·52%)
Intracranial hemorrhage	9 (1·3%)	2 (0·29%)	10 (1·75%)	1 (0·17%)
Competing risk events, *n* (%)	60 (8·68%)	42 (6·08%)	45 (7·87%)	54 (9·44%)
Drug censored[a], *n* (%)	91 (13·17%)	80 (11·58%)	81 (14·16%)	124 (21·68%)
Pair censored[b], *n* (%)		344 (49·78%)		129 (22·55%)
Median of follow-up days (IQR)	140 (32–629)	13 (6–45)	208·5 (40–669·5)	39 (9–284)
Total follow-up year	740·69	175·7	671·33	350·23
Crude incidence rate (1,000 person-years)	573·79	1,178·17	479·64	619·59
Difference in incidence rate	604·38		139·95	
Ratio of incidence rate (95% CI)	0·49 (0·44, 0·54)		0·77 (0·69, 0·87)	

Abbreviations: CI, confidence interval; IQR, interquartile range.
[a]Censored caused by holding or use of drugs.
[b]Use of drug within 30 days but categorized as controlled group owing to paired matching.

 © 2021. The Author(s).

Supplementary Table S17 Descriptive results of the severe stroke in terms of composite outcomes

	Severe stroke			
	Early treatment		Delayed treatment	
**Composite outcome**	**User**	**Nonuser**	**User**	**Nonuser**
Sample, *n*	843	843	560	560
Observed events, *n* (%)	549 (65·12%)	362 (42·94%)	354 (63·21%)	255 (45·54%)
Ischemic stroke	203 (24·08%)	106 (12·57%)	182 (32·5%)	91 (16·25%)
Gastrointestinal bleeding	164 (19·45%)	171 (20·28%)	60 (10·71%)	63 (11·25%)
Transient ischemic attack	80 (9·49%)	43 (5·1%)	46 (8·21%)	50 (8·93%)
Myocardial infarction	40 (4·74%)	14 (1·66%)	26 (4·64%)	21 (3·75%)
Hematuria	14 (1·66%)	5 (0·59%)	5 (0·89%)	6 (1·07%)
Cardiovascular death	21 (2·49%)	10 (1·19%)	15 (2·68%)	12 (2·14%)
Systemic embolism	8 (0·95%)	8 (0·95%)	5 (0·89%)	7 (1·25%)
Venous thromboembolism	12 (1·42%)	2 (0·24%)	10 (1·79%)	3 (0·54%)
Intracranial hemorrhage	7 (0·83%)	3 (0·36%)	5 (0·89%)	2 (0·36%)
Competing risk events, *n* (%)	73 (8·66%)	72 (8·54%)	41 (7·32%)	62 (11·07%)
Drug censored[a], *n* (%)	140 (16·61%)	110 (13·05%)	93 (16·61%)	131 (23·39%)
Pair censored[b], *n* (%)		275 (32·62%)		86 (15·36%)
Median of follow-up days (IQR)	63 (19–299)	21 (7–69)	88 (25·5–454·5)	44·5 (12–204·5)
Total follow-up year	584·77	262·67	477·34	312·66
Crude incidence rate (1,000 person-years)	938·83	1,378·14	741·61	815·58
Difference in incidence rate	439·31		73·97	
Ratio of incidence rate (95% CI)	0·68 (0·62, 0·75)		0·91 (0·81, 1·02)	

Abbreviations: CI, confidence interval; IQR, interquartile range.
[a]Censored caused by holding or use of drugs.
[b]Use of drug within 30 days but categorized as controlled group owing to paired matching.

 © 2021. The Author(s).

Supplementary Table S18 Descriptive results of the mild stroke in terms of effectiveness outcomes

	Mild stroke			
	Early treatment		Delayed treatment	
Effectiveness	User	Nonuser	User	Nonuser
Sample, *n*	1,325	1,325	1,198	1,198
Observed events, *n* (%)	570 (43·02%)	250 (18·87%)	447 (37·31%)	352 (29·38%)
Ischemic stroke	244 (18·42%)	105 (7·92%)	240 (20·03%)	155 (12·94%)
Transient ischemic attack	115 (8·68%)	44 (3·32%)	51 (4·26%)	62 (5·18%)
Myocardial infarction	89 (6·72%)	40 (3·02%)	62 (5·18%)	63 (5·26%)
Cardiovascular death	63 (4·75%)	30 (2·26%)	57 (4·76%)	44 (3·67%)
Systemic embolism	30 (2·26%)	17 (1·28%)	19 (1·59%)	12 (1%)
Venous thromboembolism	29 (2·19%)	14 (1·06%)	18 (1·5%)	16 (1·34%)
Competing risk events, *n* (%)	59 (4·45%)	60 (4·53%)	68 (5·68%)	84 (7·01%)
Drug censored[a], *n* (%)	312 (23·55%)	206 (15·55%)	280 (23·37%)	294 (24·54%)
Pair censored[b], *n* (%)		712 (53·74%)		306 (25·54%)
Median of follow-up days (IQR)	360 (56–907)	15 (4–73)	411·5 (82–862)	67 (11–549)
Total follow-up year	2,009·03	600·59	1,813·92	1119·64
Crude incidence rate (1,000 person-years)	283·72	416·26	246·43	314·39
Difference in incidence rate	132·54		67·96	
Ratio of incidence rate (95% CI)	0·68 (0·63, 0·74)		0·78 (0·72, 0·85)	

Abbreviations: CI, confidence interval; IQR, interquartile range.
[a]Censored caused by holding or use of drugs.
[b]Use of drug within 30 days but categorized as controlled group owing to paired matching.

 © 2021. The Author(s).

Supplementary Table S19 Descriptive results of the moderate stroke in terms of effectiveness outcomes

	Moderate stroke			
	Early treatment		Delayed treatment	
Effectiveness	User	Nonuser	User	Nonuser
Sample, *n*	723	723	602	602
Observed events, *n* (%)	356 (49·24%)	179 (24·76%)	269 (44·68%)	203 (33·72%)
Ischemic stroke	171 (23·65%)	95 (13·14%)	154 (25·58%)	94 (15·61%)
Transient ischemic attack	88 (12·17%)	57 (7·88%)	46 (7·64%)	62 (10·3%)
Myocardial infarction	38 (5·26%)	9 (1·24%)	31 (5·15%)	23 (3·82%)
Cardiovascular death	30 (4·15%)	11 (1·52%)	22 (3·65%)	13 (2·16%)
Systemic embolism	8 (1·11%)	2 (0·28%)	9 (1·5%)	6 (1%)
Venous thromboembolism	21 (2·9%)	5 (0·69%)	7 (1·16%)	5 (0·83%)
Competing risk events, *n* (%)	77 (10·65%)	70 (9·68%)	64 (10·63%)	80 (13·29%)
Drug censored[a], *n* (%)	128 (17·7%)	93 (12·86%)	103 (17·11%)	133 (22·09%)
Pair censored[b], *n* (%)		355 (49·1%)		127 (21·1%)
Median of follow-up days (IQR)	189 (36–755)	16 (6–75)	278·5 (44–805)	52 (10–382)
Total follow-up year	908·59	280·56	814·21	448·09
Crude incidence rate (1,000 person-years)	391·82	638	330·38	453·03
Difference in incidence rate	246·18		122·65	
Ratio of incidence rate (95% CI)	0·61 (0·55, 0·68)		0·73 (0·65, 0·82)	

Abbreviations: CI, confidence interval; IQR, interquartile range.
[a]Censored caused by holding or use of drugs.
[b]Use of drug within 30 days but categorized as controlled group owing to paired matching.

 © 2021. The Author(s).

Supplementary Table S20 Descriptive results of the severe stroke in terms of effectiveness outcomes

	Severe stroke			
	Early treatment		Delayed treatment	
Effectiveness	User	Nonuser	User	Nonuser
Sample, *n*	899	899	607	607
Observed events, *n* (%)	487 (54·17%)	330 (36·71%)	321 (52·88%)	249 (41·02%)
Ischemic stroke	239 (26·59%)	117 (13·01%)	204 (33·61%)	96 (15·82%)
Transient ischemic attack	182 (20·24%)	181 (20·13%)	75 (12·36%)	117 (19·28%)
Myocardial infarction	23 (2·56%)	13 (1·45%)	17 (2·8%)	15 (2·47%)
Cardiovascular death	13 (1·45%)	11 (1·22%)	7 (1·15%)	10 (1·65%)
Systemic embolism	16 (1·78%)	2 (0·22%)	5 (0·82%)	5 (0·82%)
Venous thromboembolism	14 (1·56%)	6 (0·67%)	13 (2·14%)	6 (0·99%)
Competing risk events, *n* (%)	101 (11·23%)	119 (13·24%)	59 (9·72%)	93 (15·32%)
Drug censored[a], *n* (%)	195 (21·69%)	109 (12·12%)	129 (21·25%)	148 (24·38%)
Pair censored[b], *n* (%)		306 (34·04%)		80 (13·18%)
Median of follow-up days (IQR)	68 (19–388)	20 (8–81)	99 (27–571)	50 (14–316)
Total follow-up year	742·88	365·07	597·77	412·7
Crude incidence rate (1,000 person-years)	655·56	903·93	537	603·34
Difference in incidence rate	248·37		66·34	
Ratio of incidence rate (95% CI)	0·73 (0·66, 0·8)		0·89 (0·8, 1)	

Abbreviations: CI, confidence interval; IQR, interquartile range.
[a]Censored caused by holding or use of drugs.
[b]Use of drug within 30 days but categorized as controlled group owing to paired matching.

 © 2021. The Author(s).

Supplementary Table S21 Descriptive results of the mild stroke in terms of safety outcomes

	Mild stroke			
	Early treatment		Delayed treatment	
Safety	User	Nonuser	User	Nonuser
Sample, *n*	1,383	1,383	1,358	1,358
Observed events, *n* (%)	349 (25·23%)	119 (8·60%)	330 (24·30%)	226 (16·64%)
Gastrointestinal bleeding	236 (17·06%)	97 (7·01%)	209 (15·39%)	178 (13·11%)
Hematuria	89 (6·44%)	19 (1·37%)	101 (7·44%)	44 (3·24%)
Intracranial hemorrhage	24 (1·74%)	3 (0·22%)	20 (1·47%)	4 (0·29%)
Competing risk events, *n* (%)	163 (11·79%)	117 (8·46%)	178 (13·11%)	173 (12·74%)
Drug censored[a], *n* (%)	384 (27·77%)	276 (19·96%)	343 (25·26%)	407 (29·97%)
Pair censored[b], *n* (%)		766 (55·39%)		361 (26·58%)
Median of follow-up days (IQR)	526 (158–1,019)	17 (5–143)	487 (129–907)	82 (12–580)
Total follow-up year	2,493·64	753·3	2,245·76	1,356·68
Crude incidence rate (1,000 person-years)	139·96	157·97	146·94	166·58
Difference in incidence rate	18·01		19·64	
Ratio of incidence rate (95% CI)	0·89 (0·82, 0·95)		0·88 (0·82, 0·95)	

Abbreviations: CI, confidence interval; IQR, interquartile range.
[a]Censored caused by holding or use of drugs.
[b]Use of drug within 30 days but categorized as controlled group owing to paired matching.

 © 2021. The Author(s).

Supplementary Table S22 Descriptive results of the moderate stroke in terms of safety outcomes

	Moderate stroke			
	Early treatment		Delayed treatment	
Safety	User	Nonuser	User	Nonuser
Sample, *n*	763	763	658	658
Observed events, *n* (%)	190 (24·90%)	68 (8·91%)	170 (25·84%)	96 (14·59%)
Gastrointestinal bleeding	123 (16·12%)	52 (6·82%)	102 (15·5%)	72 (10·94%)
Hematuria	52 (6·82%)	11 (1·44%)	54 (8·21%)	20 (3·04%)
Intracranial hemorrhage	15 (1·97%)	5 (0·66%)	14 (2·13%)	4 (0·61%)
Competing risk events, *n* (%)	198 (25·95%)	125 (16·38%)	124 (18·84%)	144 (21·88%)
Drug censored[a], *n* (%)	148 (19·4%)	127 (16·64%)	141 (21·43%)	207 (31·46%)
Pair censored[b], *n* (%)		406 (53·21%)		152 (23·1%)
Median of follow-up days (IQR)	376 (74–881)	16 (6–94)	424 (98–883)	55 (10–306)
Total follow-up year	1,152·07	323·82	1,021·38	445·74
Crude incidence rate (1,000 person-years)	164·92	209·99	166·44	215·37
Difference in incidence rate	45·07		48·93	
Ratio of incidence rate (95% CI)	0·79 (0·71, 0·87)		0·77 (0·69, 0·86)	

Abbreviations: CI, confidence interval; IQR, interquartile range.
[a]Censored caused by holding or use of drugs.
[b]Use of drug within 30 days but categorized as controlled group owing to paired matching.

 © 2021. The Author(s).

Supplementary Table S23 Descriptive results of the severe stroke in terms of safety outcomes

	Severe stroke			
	Early treatment		Delayed treatment	
Safety	User	Nonuser	User	Nonuser
Sample, *n*	950	950	642	642
Observed events, *n* (%)	219 (23·05%)	95 (10%)	141 (21·96%)	99 (15·42%)
Gastrointestinal bleeding	142 (14·95%)	75 (7·89%)	89 (13·86%)	71 (11·06%)
Hematuria	65 (6·84%)	18 (1·89%)	46 (7·17%)	23 (3·58%)
Intracranial hemorrhage	12 (1·26%)	2 (0·21%)	6 (0·93%)	5 (0·78%)
Competing risk events, *n* (%)	330 (34·74%)	307 (32·32%)	177 (27·57%)	218 (33·96%)
Drug censored[a], *n* (%)	229 (24·11%)	157 (16·53%)	159 (24·77%)	191 (29·75%)
Pair censored[b], *n* (%)		335 (35·26%)		89 (13·86%)
Median of follow-up days (IQR)	143 (27–585)	24 (8–112)	288·5 (59–800)	73 (16–358)
Total follow-up year	1,032·39	450·05	853·55	485
Crude incidence rate (1,000 person-years)	212·13	211·09	165·19	204·12
Difference in incidence rate	−1·04		38·93	
Ratio of incidence rate (95% CI)	1 (0·92, 1·1)		0·81 (0·73, 0·9)	

Abbreviations: CI, confidence interval; IQR, interquartile range.
[a]Censored caused by holding or use of drugs.
[b]Use of drug within 30 days but categorized as controlled group owing to paired matching.

 © 2021. The Author(s).
